# RNF138‐Mediated Ubiquitination and Degradation of NS5 Restricts Tick‐Borne Encephalitis Virus Infection

**DOI:** 10.1002/advs.75991

**Published:** 2026-06-10

**Authors:** Jialiang Sun, Weijing Yang, Hao Zhou, Shuai Li, Xin Jiang, Jianyang Gu, Wenyan Zhang

**Affiliations:** ^1^ Institute of Virology and AIDS Research Centre of Infectious Diseases and Pathogen Biology Key Laboratory of Organ Regeneration and Transplantation of the Ministry of Education State Key Laboratory for Diagnosis and Treatment of Severe Zoonotic Infectious Diseases the First Hospital of Jilin University Changchun China; ^2^ Changchun Institute of Biological Products Co., Ltd Changchun China; ^3^ Jilin Provincial Key Laboratory of Radiation Oncology & Therapy Department of Radiation Oncology & Therapy The First Hospital of Jilin University Changchun China

**Keywords:** NS5, proteasomal degradation, RNF138, TBEV, ubiquitination

## Abstract

Tick‐borne encephalitis virus (TBEV) causes severe neurological disease. However, whether host restricts its replication and the underlying mechanisms remain incompletely delineated. Here, we identify the E3 ubiquitin ligase RNF138 as an intrinsic restriction factor targeting the viral RNA‐dependent RNA polymerase (RdRp) NS5. Mechanistically, RNF138 directly interacts with the RdRp domain of NS5 through its ubiquitin‐interacting motif, catalyzes K48‐linked polyubiquitination and induces degradation of TBEV NS5. Ubiquitin‐remnant profiling and mutational analyses identify K372, K462, and K470 within NS5 as the ubiquitination sites, and mutating these residues leads to NS5 resistant to RNF138‐induced degradation. Functionally, RNF138 suppresses the replication of TBEV as well as Zika virus, a representative mosquito‐borne flavivirus, through recognizing and degrading NS5 proteins. Ectopic RNF138 reduces viral RNA levels in the brain and peripheral tissues of TBEV‐infected mice, mitigates neuroinflammatory responses, and improves survival. Notably, the antiviral activity is conserved among several mammalian RNF138 orthologs but absent in the arthropod homologs, highlighting a host‐specific antiviral adaptation. Together, these findings reveal RNF138‐mediated ubiquitination and degradation of NS5 as a mechanism of intrinsic defense against TBEV and provide a framework for exploring targeted destabilization of conserved flaviviral replicases.

## Graphical abstract

Host‐specific compatibility between RNF138‐like proteins and flavivirus NS5 determines NS5 stability. Mammalian RNF138 but not arthropod homologs recognizes and induces conserved NS5/RdRp K48‐linked ubiquitination and proteasomal degradation, thereby restricting viral replication. Ectopic RNF138 in mice attenuates TBEV‐induced pathogenesis. (Created in BioRender. Sun, J. (2026)

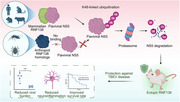

## 1 Introduction

The genus Flavivirus comprises globally distributed arthropod‐borne pathogens that impose substantial burdens on human health. Some well‐known members include tick‐borne encephalitis virus (TBEV), dengue virus (DENV), Zika virus (ZIKV), West Nile virus (WNV), and Japanese encephalitis virus (JEV). They can cause a variety of diseases ranging from hemorrhagic fever to severe neurological damage [1, 2, 3, 4]. TBEV is particularly prominent in endemic areas due to its tendency to invade the central nervous system (CNS), often leading to severe neurological complications. Currently, there are no specific antiviral drugs for TBEV, highlighting the urgent need to identify host factors that can limit its replication [5, 6]. Viral replication depends on the coordinated action of viral non‐structural (NS) proteins. Among these proteins, the non‐structural protein 5 (NS5) is highly conserved throughout the genus and serves as a key enzymatic factor. NS5 contains an N‐terminal methyltransferase (MTase) domain required for RNA capping and a C‐terminal RNA‐dependent RNA polymerase (RdRp) domain responsible for initiating viral RNA synthesis. In particular, the RdRp enzyme is absolutely essential for amplifying the viral genome [7]. Beyond these functions, NS5 also plays crucial roles in subverting host immune defenses, particularly by antagonizing interferon (IFN) signals [8, 9, 10]. Therefore, investigating how NS5 is regulated has become a major focus for understanding the pathogenesis and discovering new antiviral approaches. Such insights may extend beyond TBEV, offering broader strategies applicable to the flavivirus genus [11, 12].

The ubiquitin‐proteasome system (UPS) in eukaryotes is the main pathway for controlling protein degradation. E3 ubiquitin ligases regulate the process through substrate selection and ubiquitin chain catalysis. This polyubiquitination serves as the signal for proteasomal degradation [13, 14, 15]. Notably, this mechanism constitutes a key component of host antiviral defense. It has been reported that several viral proteins are selectively ubiquitinated and degraded through this pathway [16, 17]. Considering the essential role in flavivirus replication, NS5 is a high‐priority target for antiviral drugs development. Indeed, several host factors have been shown to antagonize flaviviruses by degrading NS proteins: the E3 ligases TRIM22 and cullin‐2, for instance, target ZIKV NS1/NS3 and NS3 for proteasomal degradation, respectively [18, 19]. Furthermore, the NS5 protein itself can be restricted by non‐E3 mechanisms, as seen with the lysosomal degradation mediated by TRIM79α or the functional inhibition by LGP2 [20, 21]. Whether E3 ubiquitin ligases recognize NS5 to mediate its proteasomal degradation remains unclear.

RNF138 is a RING finger domain E3 ligase characterized by multiple functional domains, including a canonical RING domain, tandem zinc finger motifs, and a ubiquitin‐interaction motif (UIM) [22]. It is involved in diverse fundamental biological processes, including tumor progression, DNA damage repair [23, 24, 25]. More recently, RNF138 was found to catalyze K48‐linked polyubiquitination of SMARCC1, thereby modulating inflammatory responses [26]. However, whether it targets viral proteins in antiviral defense has not been fully investigated.

Here, we identify RNF138 as an intrinsic restriction factor that targets TBEV NS5 for degradation. This antiviral action is mediated by direct binding to the RdRp domain and inducing K48‐linked polyubiquitination. As this mechanism is effective against multiple flaviviruses, it reveals a concerted strategy for broad‐spectrum innate defense centered on destabilizing the essential viral replicase. Yet the host mechanisms that regulate NS5's turnover remain poorly characterized.

## 2 Results

### 2.1 The E3 Ubiquitin Ligase RNF138 Targets TBEV NS5 for Proteasomal Degradation

**FIGURE 1:**
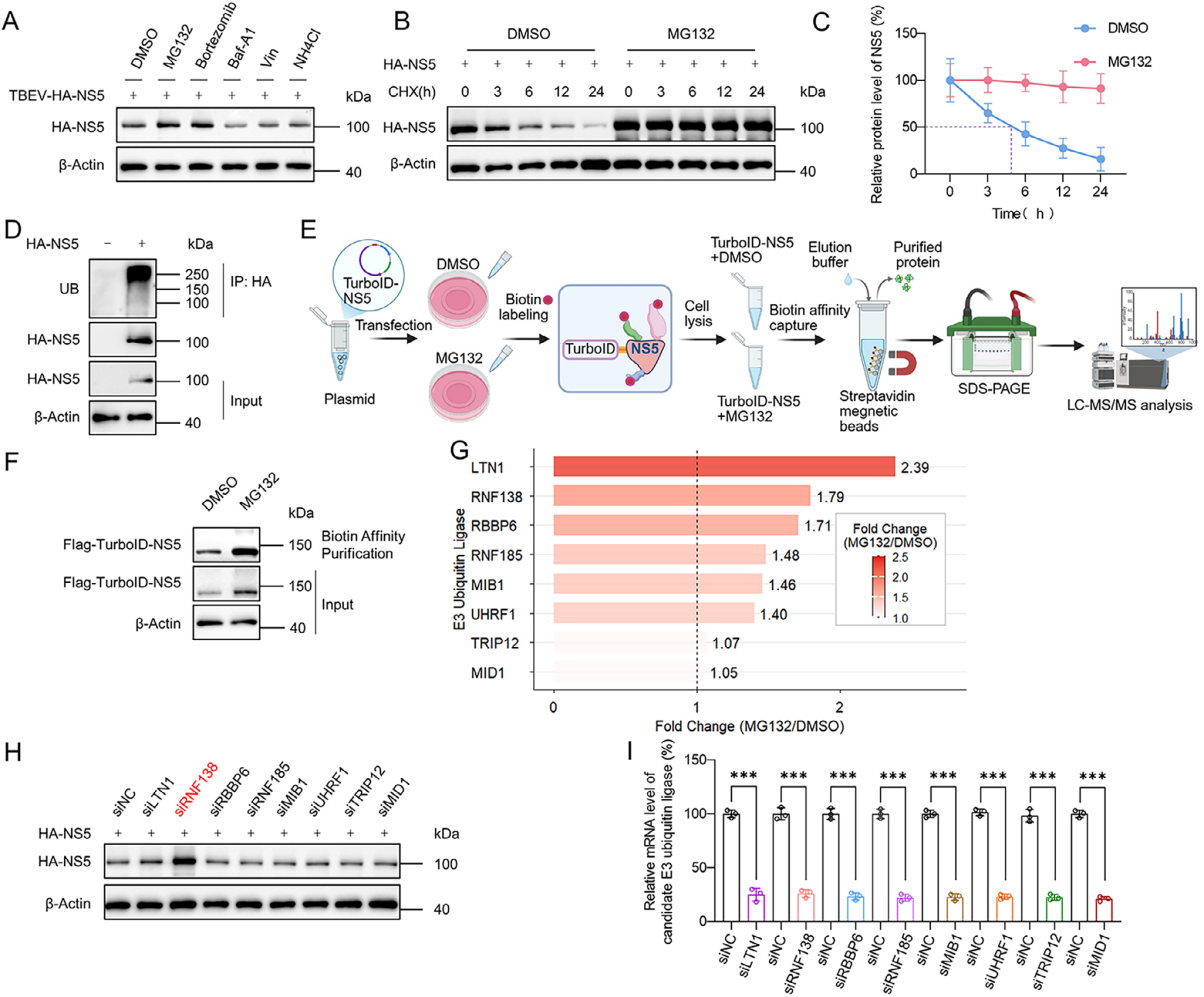
The E3 ubiquitin ligase RNF138 targets TBEV NS5 for proteasomal degradation. (A) The proteasomal inhibitors (MG132, Bortezomib) stabilize NS5. HEK293T cells expressing HA‐NS5 were treated with the indicated inhibitors for 12 h and analyzed by immunoblotting (IB). (B,C) MG132 extends the half‐life of NS5. (B) HEK293T cells expressing HA‐NS5 were treated with DMSO or MG132 (10 µm, 12 h), followed by cycloheximide (CHX, 50 µg mL^−1^) for the indicated durations. NS5 levels were assessed by IB. (C) Quantification of NS5 protein levels normalized to β‐Actin across four time points. (D) NS5 undergoes ubiquitination. HEK293T cells transfected with empty vector or HA‐NS5 were subjected to HA immunoprecipitation (IP), followed by IB with anti‐ubiquitin (Ub) antibody. (E) Workflow for TurboID‐based identification of NS5‐proximal proteins. HEK293T cells expressing Flag‐TurboID‐NS5 were treated with DMSO or MG132, followed by biotin labeling (500 µm, 10 min). Biotinylated proteins were isolated using streptavidin beads and analyzed by mass spectrum (MS) (Created with BioRender.com; agreement number: NK29359UNM). (F) Validation of the TurboID‐NS5 labeling. HEK293T cells expressing Flag‐TurboID‐NS5 were treated and labeled as in (E), followed by streptavidin affinity purification and IB. (G) Proteomic profiling of NS5‐associated E3 ubiquitin ligases. Bar graph shows fold changes (MG132/DMSO) in protein abundance of candidate E3 ubiquitin ligases identified by TurboID‐mediated proximity labeling. (H‐I) RNF138 knockdown enhances NS5 stability. (H) Based on MS screening, candidate E3 ubiquitin ligases were knocked down using siRNAs in HEK293T cells for 24 h, followed by HA‐NS5 transfection for 48 h. Lysates were analyzed by IB. (I) mRNA levels of E3 ubiquitin ligase candidates were determined by RT‐qPCR, and presented as mean ± SD. Data are mean ± SD; ***p < 0.001 by two‐tailed t‐test. Unless stated otherwise, MG132 treatment = 10 µm, 12 h. All data are representative of at least three independent experiments.

As the most conserved and functionally indispensable enzyme for RNA synthesis in flaviviruses, NS5 remains an attractive target for intrinsic antiviral regulation [7, 27, 28]. To determine whether host pathways influence NS5 stability, HA‐NS5‐expressing HEK293T cells were treated with indicated inhibitors. The proteasome inhibitors MG132 or bortezomib, both caused a marked accumulation of NS5, while inhibitors of lysosomal or autophagy‐related pathways, including bafilomycin A1 (Baf‐A1), vinblastine sulfate (Vin), and NH_4_Cl, produced minimal changes (Figure 1A). Consistent with this, cycloheximide (CHX) chase assays showed that MG132 substantially prolonged the half‐life of NS5 (Figure 1B,C). Immunoprecipitation (IP) of HA‐tagged NS5 followed by immunoblotting (IB) further demonstrated that NS5 is modified by polyubiquitin chains (Figure 1D).

To identify the E3 ubiquitin ligase mediating NS5 ubiquitination, we performed TurboID‐based proximity labeling, which is well suited for detecting weak or transient interactions that might escape classical co‐IP [29]. HEK293T cells expressing Flag‐TurboID‐NS5 were treated with biotin following MG132 or DMSO treatment, and biotinylated proteins were isolated by streptavidin affinity purification for mass spectrometry (MS) analysis (Figure 1E,F). Some candidate E3 ubiquitin ligases, including LTN1, RNF138, RBBP6, RNF185, MIB1, UHRF1, TRIP12 and MID1 showed significantly increased normalized abundance ratios (MG132/DMSO) (Figure 1G). A focused siRNA screen targeting these candidates identified RNF138 as the most relevant hit; depletion of RNF138, but not other E3 ligases, elevated NS5 protein levels, with knockdown efficiency verified by RT‐qPCR (Figure 1H,I). Lentiviral delivery of RNF138‐specific shRNA yielded similar results (Figure S1A). In contrast, RNF138 overexpression reduced NS5 abundance in a dose‐dependent manner, and this decrease was prevented by MG132 (Figure S1B,C). CHX chase experiments showed that ectopically expressed RNF138 accelerated NS5 degradation (Figure S1D,E), whereas RNF138 silencing extended the half‐life of NS5 (Figure S1F,G). These effects occurred at the protein rather than the transcript level (Figure S1H). Together, these findings indicate that RNF138 acts as a host E3 ligase that modulates NS5 abundance by promoting its proteasomal degradation.

### 2.2 RNF138 Directly Interacts with the NS5 RdRp Domain

**FIGURE 2:**
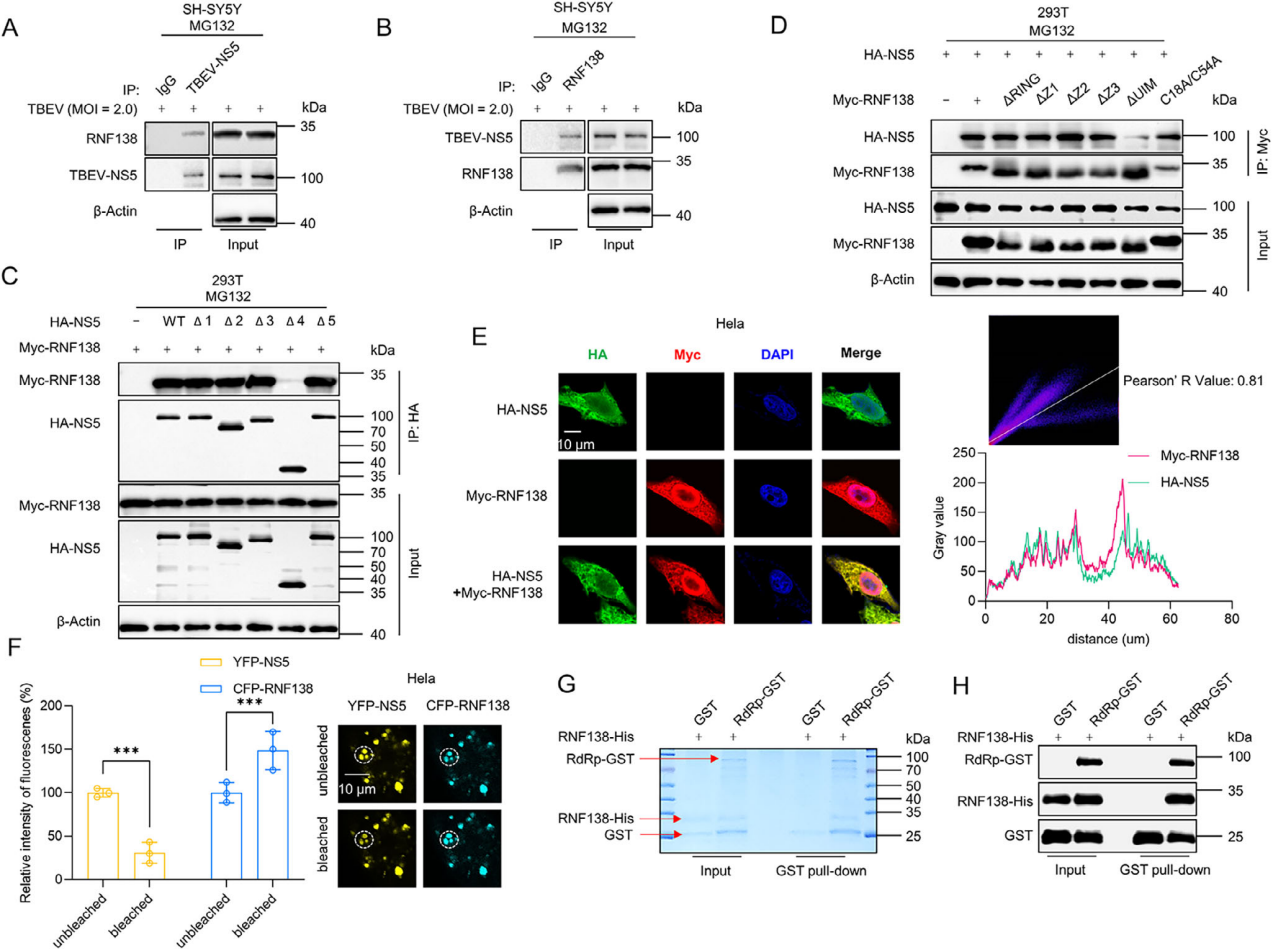
RNF138 directly interacts with the NS5 RdRp domain. (A,B) Endogenous co‐IP confirms the interaction between RNF138 and NS5 under TBEV infection conditions. SH‐SY5Y cells were infected with TBEV (MOI = 2.0) for 40 h and treated with MG132 (10 µm, 6 h) before harvest. Cell lysates were subjected to IP using anti‐NS5 antibody or control IgG (A), or anti‐RNF138 antibody or control IgG (B), followed by IB with the indicated antibodies. (C) Mapping of the NS5 domain required for RNF138 binding. HEK293T cells were co‐transfected with Myc‐RNF138 and full‐length or truncated HA‐NS5 mutants. After MG132 treatment, lysates were subjected to HA‐IP and IB. (D) Mapping of the RNF138 domain required for NS5 interaction. Cells were co‐transfected with HA‐NS5 and Myc‐RNF138 (WT, C18A/C54A, or truncations). After MG132 treatment, lysates were subjected to Myc‐IP and IB. (E) Subcellular co‐localization of NS5 and RNF138. HeLa cells co‐expressing HA‐NS5 and Myc‐RNF138 were treated with MG132, immunostained with anti‐HA (green) and anti‐Myc (red) antibodies and DAPI (blue), and imaged by confocal microscopy. Fluorescence intensity profiles along the indicated line were quantified using ImageJ. Pearson's correlation coefficient was calculated using the ImageJ Colocalization plugin. (F) FRET acceptor photobleaching demonstrates molecular proximity between NS5 and RNF138. Donor fluorescence was measured before and after acceptor photobleaching in the boxed region; FRET efficiency was calculated from the donor signal increase. (G,H) RNF138 directly binds the NS5 RdRp domain in vitro. Recombinant 6×His‐RNF138 was incubated with GST or GST‐RdRp, and complexes were pulled down with glutathione‐sepharose. (G) Coomassie staining of input and pull‐down samples; (H) IB of the same samples with anti‐His and anti‐GST antibodies. Data in (F) from three independent experiments were analyzed by paired Student's t‐test. Scale bars, 10 µm. ***p < 0.001. Unless stated otherwise, MG132 treatment = 10 µm, 12 h. All data shown are representative of at least three independent experiments.

To examine the RNF138–NS5 interaction under more physiologically relevant infection conditions, we performed endogenous co‐IP in TBEV‐infected human neuroblastoma SH‐SY5Y cells [30]. Using an anti‐NS5 antibody, endogenous RNF138 was readily detected in NS5 immunoprecipitates but not in the corresponding IgG control (Figure 2A). Conversely, IP with an anti‐RNF138 antibody efficiently pulled down NS5, whereas no specific signal was observed in the matched IgG control (Figure 2B). To map the functional regions required for their interactions, we generated a series of truncation constructs guided by the domain architectures of RNF138 and NS5 (Figure S2A,B). Co‐IP analyses identified the critical regions required for complex formation. We observed that removal of the C‐terminal RdRp domain of NS5 (shown by Δ4 mutant) nearly abolished the interaction with RNF138, indicating that RdRp is essential for the interaction (Figure 2C). On the RNF138 side, the C‐terminal UIM domain was required for association with NS5 (Figure 2D). Notably, the catalytic‐inactive mutant of RNF138 (C18A/C54A) retained the ability to bind to NS5 (Figure 2D), indicating that the catalytic activity is dispensable for the interaction. Accordingly, the RING, UIM domains and catalytic activity of RNF138 were required for NS5 degradation (Figure S2C), while NS5Δ4 mutant lacking the RdRp domain was resistant to RNF138‐mediated destabilization (Figure S2D).

To visualize RNF138‐NS5 interaction in a cellular context, we performed immunofluorescence (IF) staining in HeLa cells. Confocal microscopy revealed substantial co‐localization of HA‐NS5 and Myc‐RNF138, quantitatively supported by a high Pearson's correlation coefficient (Pearson’ *R* value: 0.81) (Figure 2E). To assess proximity at the nanometer scale, we employed FRET assay with acceptor photobleaching. Donor fluorescence significantly increased within the bleached region, yielding high FRET efficiency (Figure 2F), consistent with close proximity of RNF138 and NS5 in cells. To determine whether RNF138 and NS5 engage directly, we purified 6×His‐RNF138 in vitro which was efficiently pulled down by GST‐tagged NS5 RdRp domain but not with GST alone, as shown by Coomassie‐stained pull‐downs and IB (Figure 2G,H), demonstrating direct interaction between RNF138 and the NS5 RdRp domain. Together, RNF138 directly binds to the NS5 RdRp domain to form an enzyme–substrate complex that precedes ubiquitination and proteasomal degradation.

### 2.3 RNF138 Catalyzes K48‐Linked Polyubiquitination of NS5 at K372, K462, and K470

**FIGURE 3:**
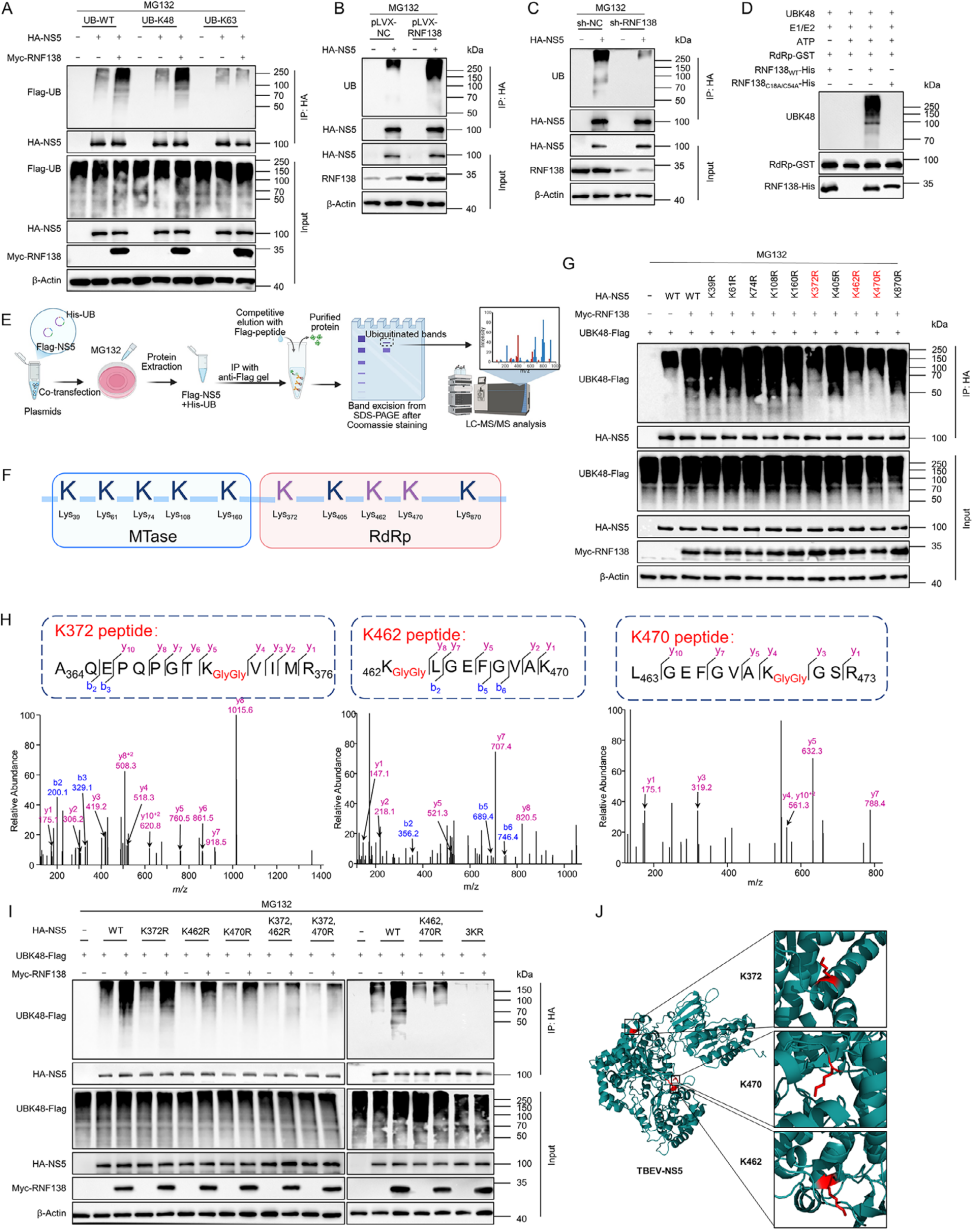
RNF138 Promotes K48‐linked Polyubiquitination of NS5 at K372, K462, and K470. (A) NS5 is preferentially modified by K48‐linked polyubiquitin chains. HEK293T cells expressing HA‐NS5 with Ub‐WT‐Flag, Ub‐K48‐Flag, or Ub‐K63‐Flag were treated with MG132 (10 µm, 12 h). HA‐IP followed by IB reveals robust K48‐linked polyubiquitination of NS5. (B) RNF138 overexpression enhances NS5 polyubiquitination. HEK293T cells co‐expressing HA‐NS5 and Myc‐RNF138 were treated with MG132 and subjected to HA‐IP and anti‐Ub IB. (C) RNF138 knockdown reduces NS5 polyubiquitination. Stable RNF138‐silenced cells expressing HA‐NS5 were treated with MG132 and analyzed by HA‐IP and anti‐Ub IB. (D) RNF138 directly catalyzes K48‐linked ubiquitination of the TBEV NS5 RdRp domain in vitro. Purified E1, E2, His‐RNF138‐WT or His‐RNF138‐C18A/C54A, GST‐tagged TBEV NS5 RdRp, K48‐linked ubiquitin, and ATP were incubated as indicated. Reaction products were analyzed by IB using an anti‐K48‐linkage‐specific antibody. (E‐F) Ubiquitin‐remnant profiling identifies candidate ubiquitination sites on NS5. (E) Workflow: HEK293T cells expressing Flag‐NS5 and 6×His‐ubiquitin were treated with MG132. Flag‐IP samples were competitively eluted and analyzed by LC‐MS/MS (Created with BioRender.com; agreement number: SA291RDS2B). (F) Ten lysine (K) residues were initially identified as candidate ubiquitination sites. (G) Mutational analysis identifies RNF138‐responsive ubiquitination sites. Cells co‐expressing Myc‐RNF138, Ub‐K48‐Flag, and HA‐NS5 (WT or individual K‐to‐R mutants) were subjected to HA‐IP and IB. (H) Representative LC‐MS/MS spectra confirming site‐specific ubiquitination at K372, K462, and K470. The spectra display K‐ε‐GG‐modified peptides. (I) The triple‐lysine mutant (3KR) abolishes RNF138‐mediated K48‐linked polyubiquitination of NS5. HEK293T cells expressing Myc‐RNF138, Ub‐K48‐Flag, and HA‐NS5 (WT or 3KR) were treated with MG132 and analyzed by HA‐IP and IB. (J) Structural mapping of NS5 ubiquitination sites. The AlphaFold3‐predicted NS5 RdRp structure is shown with K372, K462, and K470 highlighted in red. Unless stated otherwise, MG132 treatment = 10 µm, 12 h. All data shown are representative of at least three independent experiments.

We next investigated the type of ubiquitin chain assembled on NS5 by RNF138. Co‐expression of HA‐NS5 with ubiquitin variants (Ub‐WT, Ub‐K48‐only, Ub‐K63‐only) showed that RNF138 selectively promoted K48‐linked polyubiquitination of NS5, whereas no enhancement of K63‐linked modification was detected under the same conditions (Figure 3A). Consistently, RNF138 overexpression increased NS5 ubiquitination, while RNF138 knockdown reduced it (Figure 3B,C). Furthermore, in vitro ubiquitination assays with purified E1, E2, His‐RNF138, GST‐tagged NS5 RdRp domain, ubiquitin, and ATP confirmed that RNF138‐WT directly catalyzed K48‐linked ubiquitination of the NS5 RdRp domain, whereas omission of ATP or RNF138, or mutation of the RNF138 catalytic residues C18/C54, abolished this signal (Figure 3D). Consistently, NS5 ubiquitination in HEK293T cells also depended on RNF138 catalytic activity (Figure S3A,B). To map the ubiquitination sites in NS5, we performed ubiquitin‐remnant profiling by MS and identified ten candidate ubiquitination sites (lysine [K] 39, K62, K74, K108, K160, K372, K405, K462, K470, K870) (Figure 3E,F). Among single K‐to‐arginine (R) substitutions, NS5 K372R, K462R, or K470R attenuated RNF138‐mediated K48‐ubiquitination relative to wild‐type (WT) (Figure 3G). Consistent with the ubiquitination data, the single site mutants (K372R, K462R, K470R) of NS5 were partially degraded by RNF138, while the 3KR mutant was resistant to RNF138‐mediated degradation (Figure S3C). The MS results (Figure 3H) confirm ubiquitination at these specific residues (K372, K462, and K470). Accordingly, a triple mutant (3KR; K372R/K462R/K470R) nearly abolished RNF138‐mediated K48 conjugation (Figure 3I). The NS5 structure was also modeled using AlphaFold3 to visualize the spatial context of the identified ubiquitination sites (Figure 3J).

As expected, the NS5Δ4 mutant could not be ubiquitinated by RNF138 due to the loss of RNF138 binding (Figure S3D), while the RING and UIM domains of RNF138 were also essential for NS5 ubiquitination (Figure S3E). Additional loss‐ and gain‐of‐function assays corroborated RNF138‐driven K48‐linked polyubiquitination (Figure S3F,G). Because flaviviral NS5 proteins are known to antagonize IFN signaling [8], we next examined whether RNF138‐mediated NS5 degradation affects NS5‐dependent inhibition of IFN responses. As expected, NS5 markedly suppressed IFN‐β‐induced ISRE promoter activation, while co‐expression of RNF138‐WT but not C18A/C54A mutant attenuated this inhibitory effect. Notably, RNF138‐WT did not relieve the inhibitory effect mediated by the ubiquitination‐resistant NS5‐3KR mutant (Figure S3H). These results suggest that RNF138 alleviates NS5‐mediated suppression of IFN‐β‐induced ISRE activation in a manner dependent on RNF138 catalytic activity and the NS5 K372/K462/K470 ubiquitination sites.

### 2.4 RNF138 Restricts TBEV Infection in a Dose‐ and RING Domain‐Dependent Manner

**FIGURE 4:**
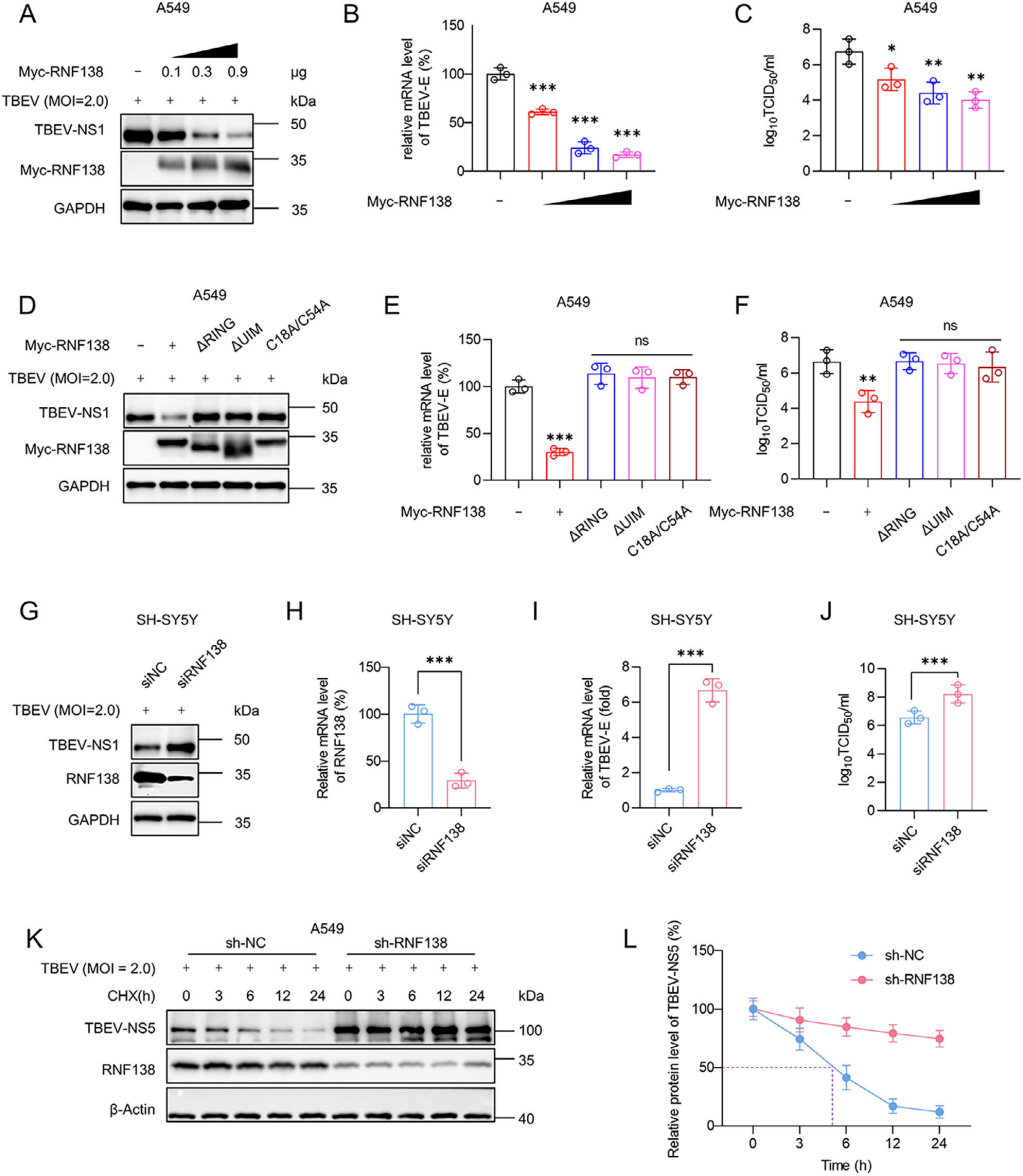
RNF138 Restricts TBEV Infection. (A–C) RNF138 inhibits TBEV infection in a dose‐dependent manner. (A) A549 cells transfected with increasing amounts of Myc‐RNF138 (0.1, 0.3, 0.9 µg) were infected with TBEV (MOI = 2.0) for 48 h. TBEV NS1 protein levels were analyzed by IB. (B) Viral RNA levels (TBEV‐E gene) and (C) supernatant viral titers were measured by RT‑qPCR and TCID_50_ assay, respectively. (D–F) The RING and UIM domains of RNF138 is essential for viral restriction. (D) A549 cells expressing RNF138 WT, ΔRING, or ΔUIM mutants were infected with TBEV (MOI = 2.0) for 48 h. TBEV NS1 protein was detected by IB. (E) Viral RNA levels and (F) viral titers were determined by RT‑qPCR and TCID_50_, respectively. (G–J) RNF138 knockdown enhances TBEV infection. (G) SH‐SY5Y cells were transfected with RNF138‐targeting siRNAs for 24 h, followed by TBEV infection (MOI = 2.0) for 48 h. TBEV NS1 protein was analyzed by IB. (H) Viral RNA levels (TBEV‐E gene) and (I) viral titers were assessed by RT‑qPCR and TCID_50_, respectively. (J) Knockdown efficiency of RNF138 was confirmed by RT‑qPCR. (K, L) RNF138 knockdown prolongs the half‐life of NS5 during TBEV infection. A549 cells stably expressing shNC or shRNF138 were infected with TBEV and then treated with CHX for the indicated times. NS5 protein levels were analyzed by IB (K), and the relative NS5 levels were quantified to β‐Actin (L). Statistical significance was determined by one‐way ANOVA (B, C, E, F), two‐tailed Student's t‐test (H‐J). Data are representative of at least three independent experiments. ns: no significance; *p < 0.05, **p < 0.01, ***p < 0.001.

To determine whether RNF138 exerts antiviral activity against TBEV through inducing NS5 degradation, we used A549 cells, a model highly susceptible to TBEV infection [31, 32], and SH‐SY5Y cells. We transfected A549 cells with increasing amounts of Myc‐RNF138 plasmid prior to viral challenge, the following IB analysis showed a progressive reduction in viral NS1 protein levels with higher RNF138 expression (Figure 4A). Consistent with this, both viral RNA levels (Figure 4B) and infectious virus production (Figure 4C) declined in a dose‐dependent manner. We further observed that deletion of the RING domain (ΔRING) or the UIM domain (ΔUIM) of RNF138 abolished its antiviral activity compared to RNF138‐WT (Figure 4D–F), indicating that the E3 ligase activity of RNF138 is essential for its antiviral function. Following knockdown of endogenous RNF138 in SH‐SY5Y and A549 cells (Figure 4J), TBEV infection was markedly enhanced (Figure 4G–I and Figure S4A–D). These loss‐of‐function assays establish endogenous RNF138 as a bona fide restriction factor against TBEV. To further determine whether endogenous RNF138 regulates NS5 stability during authentic viral infection, we performed CHX chase assays in TBEV‐infected A549 cells stably expressing shNC or shRNF138. RNF138 depletion markedly delayed NS5 turnover (Figure 4K), and quantification confirmed a prolonged NS5 half‐life upon RNF138 knockdown (Figure 4L).

We also observed the protein and mRNA levels of endogenous RNF138 were not regulated by TBEV infection (Figure S4E,F). Because many antiviral host factors are IFN‐inducible [33], we next examined whether RNF138 can be induced by IFN‐β and found no obvious change in RNF138 at either the protein or mRNA level (Figure S4G,H), while IFN‐stimulated gene 15 (ISG15) was robustly induced by IFN‐β treatment (Figure S4I), confirming that the stimulation condition was effective. Collectively, these results suggest that RNF138 expression is relatively stable and is not modulated by TBEV infection and IFN‐β.

### 2.5 RNF138 Overexpression Mitigates TBEV‐Induced Pathogenesis in Mice

**FIGURE 5:**
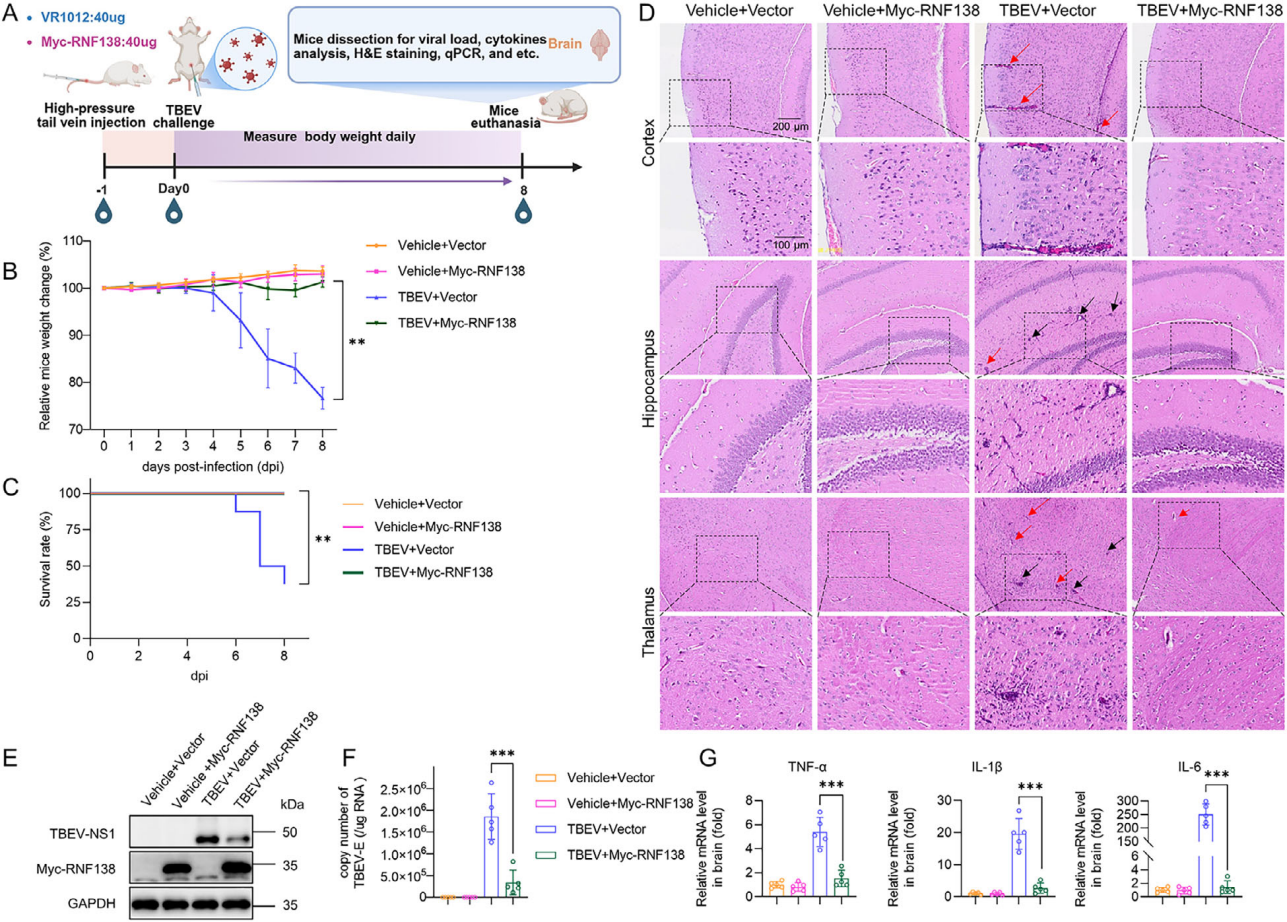
RNF138 Overexpression Attenuates TBEV Pathogenicity in a Mouse Model. (A) Schematic of the hydrodynamic plasmid delivery and TBEV infection protocol in BALB/c mice. Mice were injected with 40 µg of control or RNF138‐expressing plasmid and subsequently infected intraperitoneally with TBEV (10^3^ PFU per mice). The experimental timeline is indicated. (Created with BioRender.com; agreement number: WY291RC9UM). (B) Body weight changes of mice in each group were monitored daily following TBEV infection. Data are presented as mean ± SD. (C) Survival curves were generated using the Kaplan‐Meier method test and compared by log‐rank test (*n* = 8 per group). (D) Representative H&E‐stained sections of the hippocampus, cortex, and thalamus from mouse brains. Insets (dashed boxes) are shown below at higher magnification. Red arrows indicate perivascular cuffing; black arrows indicate necrotic foci. Scale bars: 200 µm (overview), 100 µm (magnified views). (E) The expression of Myc‐RNF138 in the brain tissues was detected by IB. (F) The viral RNA load in mice brain tissues was determined by RT‐qPCR assay of the TBEV‐E gene. Data are presented as viral genome copies per microgram of total RNA (*n* = 5 per group). (G) The mRNA expression levels of pro‐inflammatory cytokines in brain tissues were assessed by RT‐qPCR (*n* = 5 per group). Data in (F, G) are shown as mean ± SD. Each dot represents one mouse. Statistical significance was determined by two‐way ANOVA (B), log‐rank test (C), or one‐way ANOVA (F, G). *p < 0.05, **p < 0.01, ***p < 0.001.

To evaluate the antiviral potential of RNF138 in vivo, BALB/c mice were hydrodynamically injected with plasmids encoding Myc‐RNF138 or empty vector to achieve transient overexpression, followed by intraperitoneal challenge with TBEV or vehicle (Figure 5A). RNF138 overexpression protected TBEV‐infected mice from weight loss and conferred a strong survival advantage without obvious side effects (Figure 5B,C). We also examined the neuropathology of brain sections from mice and found TBEV+Vector group showed severe encephalitic lesions, including substantial inflammatory cell infiltration, necrotic foci, and pronounced perivascular cuffing in regions such as the hippocampus, thalamus, and cortex (Figure 5D), which align with previous reports from mouse models of flavivirus encephalitis [34, 35]. Notably, ectopic RNF138 reduced encephalitic lesions caused by TBEV infection and significantly lowered the brain viral burden (measured by TBEV‐E gene copies), consistent with the improvements in clinical and pathological outcomes (Figure 5D–F). As expected, uninfected control groups showed no or minimal pathology (Figure 5D). Furthermore, RNF138 overexpression reduced mRNA levels of several pro‐inflammatory mediators, including TNF‐α, IL‐1β, and IL‐6 (Figure 5G).

To further define the tissue distribution of hydrodynamically delivered RNF138, we measured exogenous Myc‐RNF138 transcripts in the liver, spleen, kidney, and brain of mice. Myc‐RNF138 expression was highest in the liver and lowest in the brain (Figure S5A), consistent with the expected tissue preference of hydrodynamic plasmid delivery [36, 37]. We next examined the tissue distribution of TBEV burden in infected mice receiving control vector or Myc‐RNF138 plasmid. Among the brain, liver, spleen, and kidney, TBEV RNA levels were highest in the brain (Figure S5B), indicating that the brain represents the major site of viral accumulation. However, RNF138 overexpression reduced TBEV‐E RNA levels in the indicated tissues, as well as the TBEV‐E genome copy number in blood samples (Figure S5B,C), suggesting that ectopic RNF138 may exert antiviral effects in peripheral tissues, the CNS and circulation. We then assessed the cellular distribution of ectopic RNF138 in brain sections by double IF staining. In mice receiving Myc‐RNF138 plasmid, Myc signals were detectable in the brain and showed prominent overlap with the endothelial marker CD31 [38], although a small fraction of Myc‐positive signals was not clearly associated with CD31 (Figure S5D). In contrast, Myc signals showed little overlap with the neuronal marker NeuN (Figure S5E) [39]. These data indicate a predominantly vascular‐associated distribution of ectopic RNF138 in the brain, with minimal neuronal localization. Together, these findings demonstrate that ectopic RNF138 protects against TBEV infection in vivo. Given the liver‐biased expression after hydrodynamic delivery and the vascular‐associated RNF138 signal in the brain, this protection may involve both peripheral antiviral effects and CNS vascular‐associated restriction.

### 2.6 RNF138 Exerts Antiviral Potential against Multiple Flaviviruses

**FIGURE 6:**
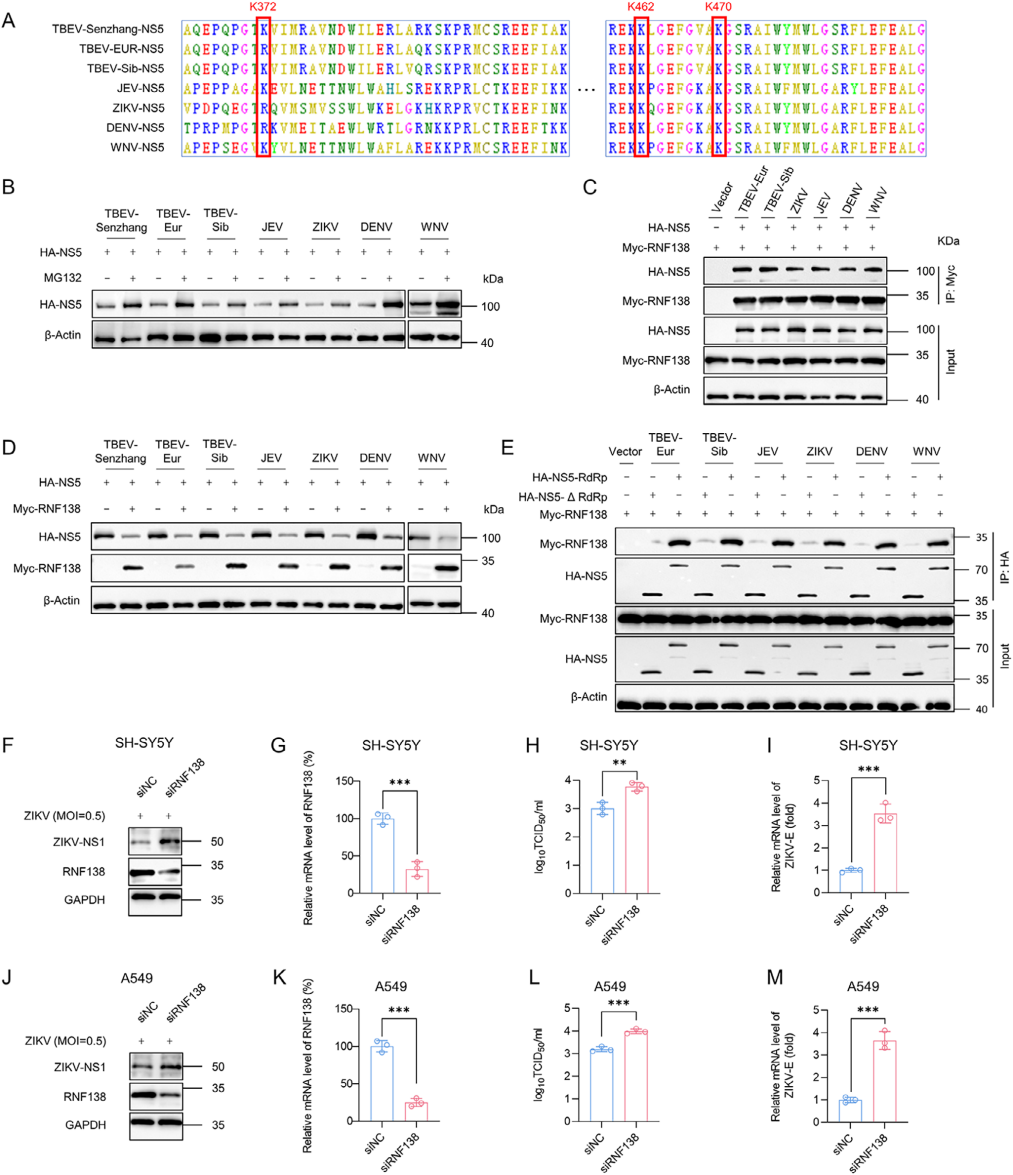
RNF138 exerts antiviral potential against multiple flaviviruses. (A) Sequence alignment of the NS5 protein from arthropod‐borne flaviviruses. Key ubiquitination acceptor sites (K372, K462, K470 in TBEV NS5) and their conserved lysine residues in other flaviviruses are highlighted. (B) Proteasomal inhibition stabilizes NS5 proteins across multiple flaviviruses. HEK293T cells expressing HA‐tagged NS5 from the indicated flaviviruses were treated with MG132 (10 µm, 12 h) and analyzed by IB. (C) RNF138 interacts with NS5 proteins from multiple flaviviruses. HEK293T cells were co‐transfected with Myc‐RNF138 and HA‐tagged NS5 from the indicated flaviviruses. Cell lysates were subjected to Myc‐IP, followed by IB. (D) RNF138 promotes degradation of NS5 from diverse flaviviruses. HEK293T cells co‐expressing Myc‐RNF138 and HA‐tagged NS5 from the indicated flaviviruses were harvested at 48 h post‐transfection and analyzed by IB. (E) The RdRp domain contributes to RNF138 binding across multiple flaviviral NS5 proteins. HEK293T cells were co‐transfected with Myc‐RNF138 and HA‐tagged RdRp domains or the corresponding ΔRdRp truncation mutants from the indicated flaviviruses. Cell lysates were subjected to Myc‐IP and analyzed by IB. (F–I) RNF138 knockdown enhances Zika virus (ZIKV) infection in SH‐SY5Y cells. (F) SH‐SY5Y cells transfected with RNF138‐targeting or control siRNAs for 24 h were infected with ZIKV (MOI = 0.5) for 48 h. ZIKV NS1 protein levels were analyzed by IB. (G) Knockdown efficiency of RNF138 was confirmed by RT‑qPCR. (H) Viral RNA levels (ZIKV‐E gene) and (I) viral titers in the supernatant were measured by RT‑qPCR and TCID_50_ assay, respectively. (J–M) RNF138 knockdown enhances ZIKV infection in A549 cells. (J) Cells transfected with RNF138‐targeting or control siRNAs for 24 h were infected with ZIKV (MOI = 0.5) for 48 h. ZIKV NS1 protein levels were analyzed by IB. (K) Knockdown efficiency was confirmed by RT‑qPCR. (L) Viral RNA levels (ZIKV‐E gene) and (M) viral titers were measured by RT‑qPCR and TCID_50_ assay, respectively. Data are presented as mean ± SD; **p < 0.01, ***p < 0.001 by two‐tailed Student's t‐test. All data are representative of at least three independent experiments.

Flaviviruses, including both tick‐borne (like TBEV) and mosquito‐borne (like ZIKV) members, cause global therapeutic challenge [40, 41]. An ideal antiviral strategy would have activity across this broad group of pathogens [42]. We next explored whether the RNF138‐mediated degradation of TBEV NS5 might also apply to other flaviviruses. Interestingly, sequence alignment revealed that the three key ubiquitination sites (K372, K462, K470) identified here are highly conserved in the major flavivirus NS5 (Figure 6A). This strong structural preservation pointed to a shared vulnerability. We first found that the proteasome inhibitor MG132 stabilized NS5 protein levels across indicated flaviviruses (Figure 6B), indicating that the UPS commonly regulates flavivirus NS5. Co‐IP analysis showed that RNF138 pulled down NS5 proteins from the indicated flaviviruses (Figure 6C). RNF138 also reduced the abundance of the indicated flaviviral NS5 proteins (Figure 6D). Furthermore, RNF138 efficiently interacted with RdRp domains but not Δ4 (ΔRdRp) truncation mutants (Figure 6E). These data suggest that RNF138 interacts with multiple flaviviral NS5 proteins and that the RdRp region contributes to this interaction. To assess the broad antiviral activity of RNF138, we tested its effect on ZIKV infection in both SH‐SY5Y and A549 cells. RNF138 knockdown resulted in an increased level in viral NS1 protein, viral RNA, and infectious virus production in both cells (Figure 6F–M). Together, these findings suggest that RNF138 has antiviral potential against multiple flaviviruses, possibly through recognition and destabilization of NS5.

### 2.7 RNF138‐Mediated Recognition of TBEV NS5 and Antiviral Restriction Are Conserved across Mammalian Species

To investigate whether RNF138‐mediated recognition of TBEV NS5 is conserved in other vertebrate hosts, we compared RNF138 sequences from human, mouse, rhesus macaque, and bovine. Sequence alignment showed that these RNF138 orthologs are highly conserved, with the catalytic cysteines in the RING domain, C18 and C54, preserved across all four species (Figure S6A). These RNF138 orthologs all reduced the protein level of TBEV NS5 and interacted with it (Figure S6B,C). Mouse RNF138 (mRNF138) WT promoted K48‐linked ubiquitination and degradation of TBEV NS5, whereas the catalytic mutant mRNF138 C18A/C54A did not (Figure S6D,E). To further examine the functional relevance of this conservation, we performed rescue experiments in A549 sh‐RNF138 cells. Reconstitution with shRNA‐resistant human RNF138 or mRNF138 reduced viral NS1 protein levels, viral RNA levels, and infectious viral titers, whereas the corresponding catalytic mutants did not (Figure S6F–H). Together, these findings demonstrate that the tested mammalian RNF138 orthologs retain conserved NS5 recognition and E3 ligase‐dependent antiviral activity against TBEV.

### 2.8 Arthropod RNF138 Homologs Do Not Interact with or Destabilize Flaviviral NS5

**FIGURE 7:**
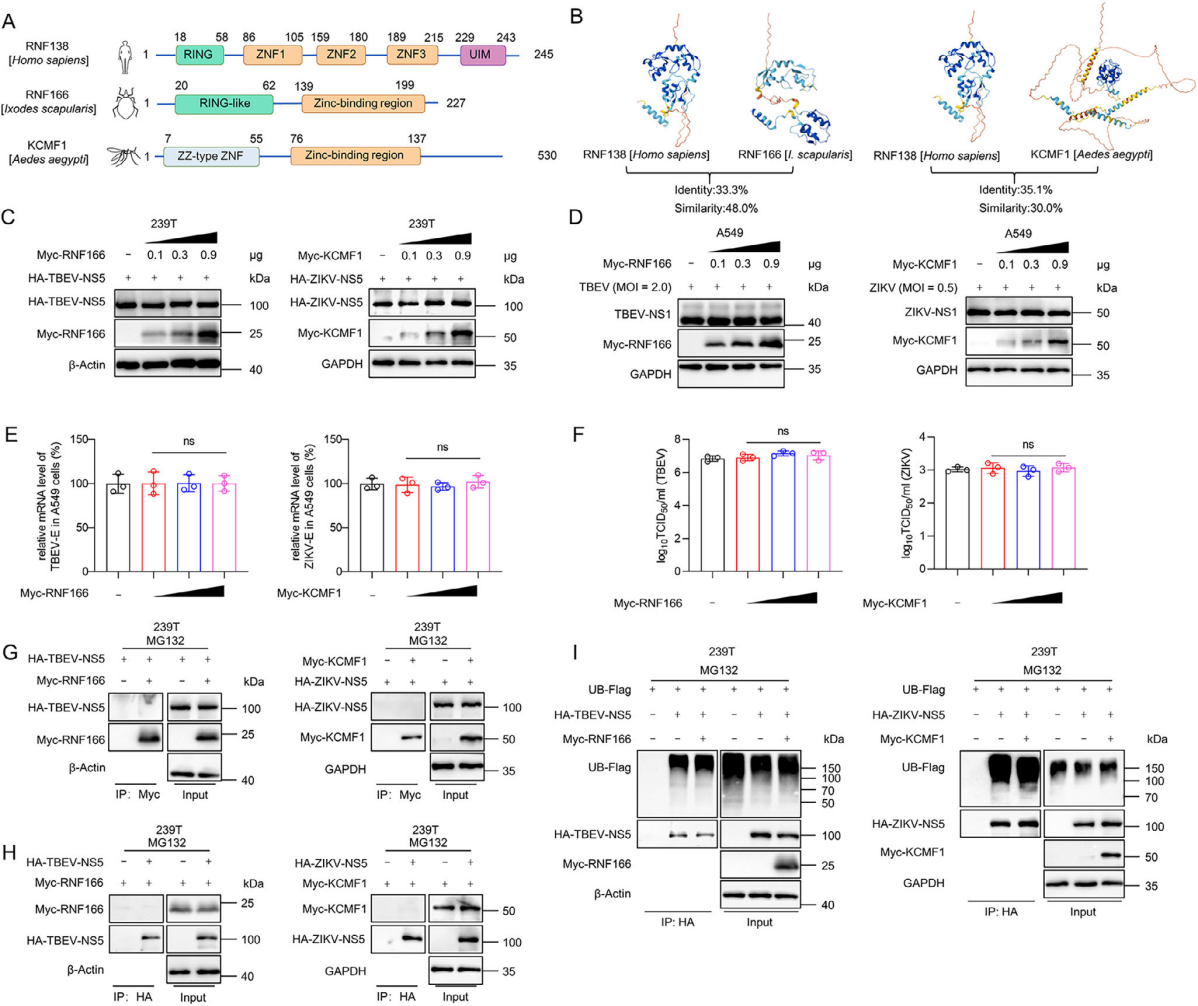
Arthropod RNF138 homologs do not interact with or destabilize flaviviral NS5. (A) Domain architecture comparison of human RNF138, *I. scapularis* RNF166, and *A. aegypti* KCMF1. Predicted domain annotations were derived from NCBI conserved domain search. Human RNF138 contains a canonical RING, three zinc finger motifs (ZNF1‐3), and a UIM domain. Tick RNF166 shows RING‐like and zinc‐binding regions, while mosquito KCMF1 contains ZZ‐type zinc finger and zinc‐binding regions. (B) Structural models of human RNF138 and arthropod RNF138 homologs predicted by AlphaFold3. The predicted structures of human RNF138 and tick RNF166 (left), and human RNF138 and mosquito KCMF1 (right) are shown. (C) Arthropod RNF138 homologs do not promote NS5 degradation. HEK293T cells were co‐transfected with increasing amounts of Myc‐RNF166 (0.1, 0.3, 0.9 µg) and HA‐TBEV‐NS5 (left), or co‐transfected with Myc‐KCMF1 (0.1, 0.3, 0.9 µg) and HA‐ZIKV‐NS5 (right), followed by IB. (D‐F) Arthropod RNF138 homologs do not inhibit corresponding flavivirus replication. A549 cells were transfected with increasing amounts of Myc‐RNF166 or Myc‐KCMF1 plasmids, followed by TBEV (MOI = 2.0) or ZIKV (MOI = 0.5) infection 24 h later. (D) Viral NS1 protein levels were analyzed by IB. (E) Viral RNA levels (TBEV‐E or ZIKV‐E gene) were quantified by RT‐qPCR. (F) Viral titers were determined by TCID_50_ assay. (G, H) Arthropod RNF138 homologs do not interact with corresponding NS5 proteins. HEK293T cells co‐expressing HA‐TBEV NS5 and Myc‐RNF166, or HA‐ZIKV NS5 and Myc‐KCMF1, were treated with MG132 (10 µm, 12 h). Lysates were subjected to Myc‐IP (G) or HA‐IP (H), followed by IB. (I) Arthropod RNF138 homologs do not enhance NS5 ubiquitination. HEK293T cells co‐expressing HA‐TBEV‐NS5, UB‐Flag, and Myc‐RNF166, or HA‐ZIKV‐NS5, UB‐Flag, and Myc‐KCMF1, were treated with MG132 (10 µm, 12 h), followed by HA‐IP and IB. Data are presented as mean ± SD; ns, no significance, by one‐way ANOVA (E, F). All data shown are representative of at least three independent experiments.

The potent restriction of TBEV by human RNF138 prompted us to explore the evolutionary aspects of this host‐pathogen interaction [43]. As arthropod‐borne viruses, TBEV and ZIKV are maintained through transmission cycles involving arthropod vectors and vertebrate hosts. We therefore asked whether RNF138 homologs from arthropod vectors possess similar NS5‐targeting activity. A BLAST‐based search identified RNF166 (NCBI accession number: XP_029838554.1) as the closest *Ixodes scapularis (I. scapularis)* homolog of human RNF138. In addition, because *Aedes aegypti (A. aegypti)* is a major mosquito vector responsible for the transmission of several medically important arboviruses, including ZIKV, we selected the *A. aegypti* RNF138 homolog KCMF1 (NCBI accession number: XP_001656420.2) for further analysis. Domain comparison showed that human RNF138, tick RNF166, and mosquito KCMF1 differ substantially in their overall domain organization and sequence features (Figure 7A,B).

We first examined whether these arthropod homologs regulate NS5 protein stability and found that increasing expression of tick RNF166 did not reduce TBEV NS5 abundance. Similarly, ectopic mosquito KCMF1 failed to decrease ZIKV NS5 levels (Figure 7C). Accordingly, RNF166 and KCMF1 did not reduce the replication of TBEV and ZIKV, respectively, by detecting NS1 protein accumulation, viral RNA levels, or infectious virus titers (Figure 7D–F). To dissect the reason why RNF166 and KCMF1 lack antiviral activity, we performed reciprocal co‐IP assays. Neither Myc‐IP of RNF166/KCMF1 nor HA‐IP of the corresponding NS5 protein pulled down the reciprocal partner. Similarly, no detectable interaction was observed between ZIKV NS5 and mosquito KCMF1 under the same experimental conditions (Figure 7G,H). Consistently, neither RNF166 nor KCMF1 enhanced ubiquitination of the corresponding NS5 proteins (Figure 7I). Together, these results indicate that the RNF138‐like antiviral activity observed in mammalian cells is not conserved in the examined arthropod homologs RNF166 and KCMF1, which likely explains the transmission and proliferation of flaviviruses in arthropod vectors.

## 3 Discussion and Conclusion

**FIGURE 8:**
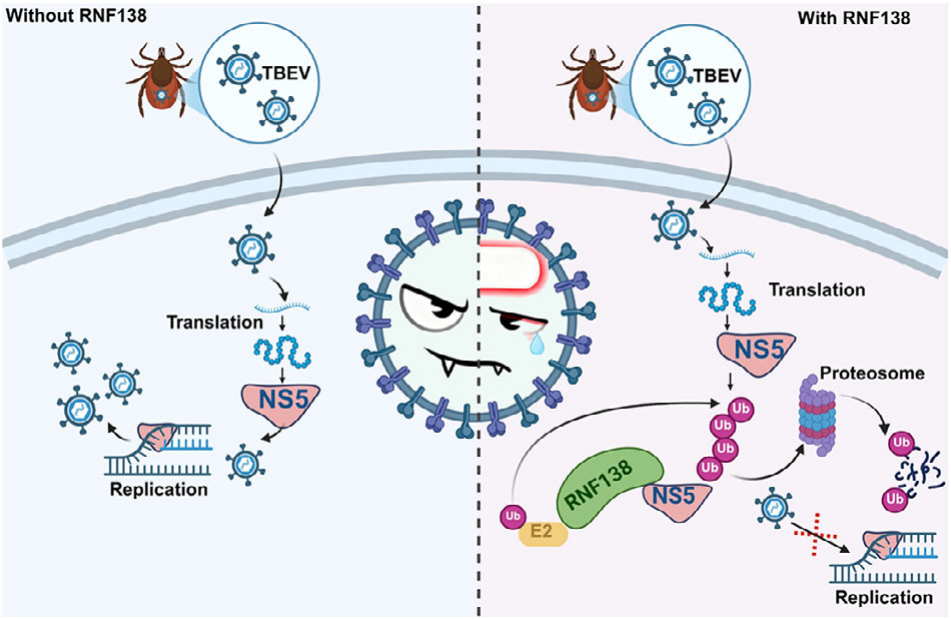
Schematic diagram of E3 ligase RNF138 mediating ubiquitination and proteasomal degradation of TBEV NS5 to inhibit viral replication (Created With BioRender.com; Agreement Number: TN291RCX30).

As a central hub for viral RNA synthesis, the flavivirus NS5 protein represents a critical target that host defenses may exploit to restrict infection [44, 45, 46, 47]. In this study, we identify that the E3 ubiquitin ligase RNF138 degrades NS5, revealing a previously unrecognized mechanism in the host's constraint on the replication of flaviviruses. Our findings demonstrate that RNF138 directly binds to the NS5 RdRp domain and catalyzes K48‐linked polyubiquitination, a reaction further supported by the in vitro ubiquitination assay (Figure 3D), thereby facilitating proteasomal degradation of NS5 and restricting TBEV replication (Figure 8).

Specific ubiquitin sites (K372, K462, and K470) within the NS5 RdRp domain were also identified. Structural modeling indicates that these targeted lysine residues are located in solvent‐accessible regions of NS5 (Figure 3J), which supports their role as ubiquitin sites. Notably, these residues are relatively conserved across several major pathogenic flaviviruses, suggesting they are subject to similar UPS regulatory mechanisms (Figure 6). Consistent with this, RNF138 interacted with NS5 proteins from multiple flaviviruses, and the RdRp region contributed substantially to this interaction. These findings support the possibility that conserved features within the NS5 RdRp domain may underlie a shared vulnerability to RNF138‐mediated destabilization [48]. As expected, we confirmed that RNF138 inhibits ZIKV replication. Such broad‐spectrum antiviral characteristic of RNF138 also distinguishes it from more specialized restriction factors such as certain IFITM proteins [49]. Future surveillance of naturally occurring substitutions at these lysine residues may help determine whether variation at these sites affects RNF138 sensitivity, viral fitness, or pathogenicity.

An important finding is that although the RING domain is necessary for the ubiquitin transfer that leads to protein degradation [50, 51], the binding of RNF138 to NS5 does not depend on its catalytic activity, substrate binding is preserved even when RNF138's catalysis is inactivated. This mode shows that substrate recognition can be separated from catalytic activation. Given the UIM domain's essential role, we speculate that these auxiliary motifs within RNF138 are involved in coordinating or stabilizing the ubiquitination machinery during the modification of NS5 [23, 52]. This pattern may enable RNF138 to adapt to the sequence variations of NS5 in different flaviviruses while maintaining its antiviral role.

RNF138 orthologs from mouse, rhesus macaque, and bovine also retained the capacity to interact with TBEV NS5 and reduce its abundance, suggesting that the RNF138–NS5 axis may represent a conserved host–virus interface in several mammalian species. In contrast, the tick homolog RNF166 and the mosquito homolog KCMF1 failed to bind and degrade the corresponding flaviviral NS5 proteins, or inhibit viral replication, indicating that mammalian RNF138‐induced NS5 degradation is not conserved in the examined arthropod homologs. This functional divergence may reflect distinct evolutionary pressures between vertebrate hosts and arthropod vectors [5]. In arthropod vectors, RNF138 orthologs lost the ability to degrade NS5 may be compatible with persistent or non‐cytolytic viral maintenance, although this possibility requires further investigation in authentic vector cells and in vivo vector models [53].

TBEV neuropathogenesis involves viral replication and inflammatory injury in the CNS [5]. Our in vivo findings demonstrate that transient RNF138 overexpression can mitigate TBEV‐induced disease, extending the antiviral role of RNF138 beyond cell culture. The tissue‐distribution analysis showed that hydrodynamic plasmid delivery produced a predominantly peripheral, liver‐biased RNF138 expression pattern, with limited vascular‐associated signal in the CNS. Whether neuron‐targeted RNF138 expression can further restrict TBEV replication within the CNS remains an interesting question for future studies using targeted delivery or cell type‐specific models.

While this study establishes RNF138‐mediated NS5 degradation as a potent antiviral mechanism, it also opens several important questions for future research. First, although we did not observe marked changes in RNF138 abundance after TBEV infection or IFN‐β stimulation, we cannot exclude the possibility that flaviviruses may use more subtle countermeasures to modulate RNF138 activity rather than its expression. Future studies are warranted to explore such potential regulatory mechanisms and determine whether they influence RNF138 antiviral activity in different cellular contexts or in vivo. Second, translating these findings into clinical applications remains challenging. Approaches such as naked DNA delivery are still limited by safety and efficacy considerations [16]. Therefore, safer and more feasible strategies, including small molecules that modulate RNF138 activity or stability, require further investigation. Recent studies using viral protein degraders or small‐molecule modulation of antiviral E3 ligase pathways support the feasibility of targeted viral protein destabilization as an antiviral strategy [54, 55]. Finally, we need to evaluate this mechanism within a more clinically relevant framework. Future studies should be based on clinical samples and clinical research, focusing on exploring the potential of RNF138 as a biomarker for predicting the severity or prognosis of the disease in patients [56]. Therefore, answering these questions will clarify RNF138's role in intrinsic immunity and may enable new antiviral strategies that trigger targeted viral proteins degradation.

Collectively, our work identifies RNF138 as a host restriction factor against TBEV infection and shows broad‐spectrum antiviral potential against flaviviruses. Mechanistically, RNF138 promotes proteasomal degradation of NS5 through ubiquitination of relatively conserved lysine residues within the RdRp domain. These findings provide new insight into how the UPS contributes to flavivirus restriction and may inform future strategies aimed at targeted destabilization of viral proteins.

## 4 Materials and Methods

### 4.1 Plasmid Construction

Gene sequences encoding the NS5 protein from TBEV subtypes (Senzhang, Siberian [Sib], and European [Eur]) and other flaviviruses including ZIKV, JEV, DENV, and WNV were synthesized by Comate BioScience (Changchun, China) and subcloned into the VR1012 vector with an N‐terminal HA tag, based on published sequences with verified NCBI accession numbers (GenBank IDs, provided in Table S1). The human RNF138 gene was amplified by PCR from cDNA derived from SH‐SY5Y cells and cloned into VR1012 between the EcoRI and BamHI sites, incorporating an N‐terminal Myc tag. Mutants and truncations of TBEV‐Senzhang HA‐NS5 and Myc‐RNF138 were generated using site‐directed mutagenesis or overlap extension PCR. Plasmids encoding shRNA‐resistant RNF138, Myc‐tagged vertebrate RNF138 orthologs, based on reference RNF138 amino acid sequences from UniProt (provided in Table S2), and flaviviral NS5 derivatives were generated by Shanghai Sangon Biotech (Shanghai, China) through commercial gene synthesis, subcloning, or mutagenesis services. For shRNA‐resistant RNF138 constructs, synonymous substitutions were introduced into the shRNA‐targeted region without altering the encoded amino acid sequence. NS5 truncation, RdRp‐domain, and amino acid substitution constructs were generated according to the designed sequences and cloned into the VR1012 vector. Plasmids expressing Myc‐tagged versions of *Ixodes scapularis* RNF166 (Cat. No. G75519) and *Aedes aegypti* KCMF1 (Cat. No. G109299) were obtained from Wuhan Miaolingbio (Wuhan, China). Both genes were cloned into the pCAGGS‐EcoRI‐Myc vector (Cat. No. G75592), which also served as the empty vector control in this study.

For proximity labeling assays, the coding region of TBEV‐Senzhang NS5 was inserted into the EcoRI and XbaI sites of the Flag‐TurboID vector (Addgene, #124646, USA). For immunofluorescence and FRET assays, the coding sequences for TBEV‐Senzhang NS5 and human RNF138 were individually cloned into the pcDNA3‐YFP (Addgene, #13033, USA) and pECFP‐C1 (BD Biosciences Clontech, 6076‐1, USA) vectors, respectively. For recombinant protein expression in *Escherichia coli*, the DNA fragments encoding the RdRp domain of TBEV NS5 and full‐length human RNF138 were amplified by PCR with primers containing 15–20 bp homology arms. These fragments were subsequently cloned into the linearized pGEX‐6P‐1 (Cytiva, 28‐9546‐49, USA) and pET‐28a (Novagen, 69864‐3, USA) vectors, respectively, using the pEASY‐Basic Seamless Cloning and Assembly Kit (TransGen Biotech, CU201‐02, China), resulting in N‐terminal GST‐ and dual 6×His‐ tagged fusion proteins. All primers and synthesized gene fragments used for plasmid construction are listed in Table S2. All other plasmids were available from our laboratory stock.

### 4.2 Cell Culture, Viruses, and Titrations

HEK293T (CRL‐11268), A549 (CCL‐185), Vero‐E6 (CRL‐1586), and HeLa (CRM‐CCL‐2) cells were obtained from the American Type Culture Collection (ATCC, Manassas, VA, USA). Cells were maintained in Dulbecco's Modified Eagle's Medium (DMEM; Gibco, C11995500BT, USA) supplemented with 10% fetal bovine serum (FBS; PAN Seratech, ST30‐3302, Germany) at 37 °C under 5% CO_2_. Human neuroblastoma SH‐SY5Y cells (CRL‐2266; ATCC) were cultured in MEM (Gibco, 11095080, USA)/F‐12 medium (Gibco, C11765500BT, USA) containing 10% FBS under the same conditions. All cell lines used in this study, confirmed to be mycoplasma‐free by a stain assay kit (Beyotime, C0296, China), were maintained under standard conditions.

The TBEV Senzhang strain (GenBank: AY182009.1) used in mouse infection model was provided by Changchun Institute of Biological Products Co., Ltd. The virus was propagated in Vero‐E6 cells cultured in DMEM with 10% FBS. All experiments involving TBEV infection were performed under Biosafety Level 3 (BSL‐3) laboratory. ZIKV infectious clone was constructed by reversed genetics according to the sequences (GenBank: KU963796.1) by our laboratory, then was transfected into HEK293T cells to produce viruses, which were amplified in Vero‐E6 cells.

Viral titers were determined by TCID_50_ assay in A549 and SH‐SY5Y cells. In brief, confluent monolayers in 96‐well plates were inoculated with 100 µL of serially diluted virus (10^−1^ to 10^−10^). After 3–5 d of incubation, cytopathic effects (CPE) were recorded. The TCID_50_ was calculated using the Reed–Muench method.

### 4.3 Transfection and Infection

DNA transfections in HEK293T, A549, and HeLa cells were performed using Lipofectamine 3000 Reagent (Invitrogen, L3000‐008, USA). The siRNA transfections were conducted with Lipofectamine RNAiMAX Reagent (Invitrogen, 13778150, USA), following the manufacturer's protocols. For viral infection, cells at 60–70% confluence in multi‐well plates were incubated with TBEV (MOI = 2.0) or ZIKV (MOI = 0.5) at 37°C for 4–6 h. After infection, cells were washed with PBS (Corning, 21–040, USA) and cultured in fresh medium. The cells were harvested 48 h post‐infection for subsequent experiments.

### 4.4 SiRNA and shRNA Construction

Specific siRNAs of host genes and RNF138‐specific shRNA were purchased from Comate Bioscience (Changchun, China), the corresponding sequences were listed in Table S3.

### 4.5 Immunoblotting (IB), Immunoprecipitation (IP), and Antibodies

Proteins were extracted from both cultured cells and mouse brain tissues with RIPA lysis buffer. The protein concentrations of mice tissue lysates were quantified using a BCA assay kit (Beyotime, P0012S, China) to normalize loading. Subsequently, all samples were diluted with loading buffer, heat‐denatured, and resolved on 12% SDS‐PAGE gels. Proteins were transferred to PVDF membranes and blocked with blocking buffer (Epizyme Biotech, PS108, China) for 0.5 h at 24°C. Membranes were then incubated overnight at 4°C with primary antibodies, followed by incubation with HRP‐conjugated secondary antibodies (Jackson ImmunoResearch, 115‐035‐062 [anti‐mouse] and 111‐035‐045 [anti‐rabbit], USA) for 1 h at 24°C. Protein bands were visualized using the SuperLumia ECL Kit (Proteintech, B500022, USA). At least three independent experiments were performed.

For IP, to prevent NS5 degradation, cells were treated with MG132 (10 µm) (Abcam, ab141003, UK) for 12 h and then harvested and resuspended in lysis buffer containing MG132 (10 µm) and protease inhibitors (Roche, 11697498001, Switzerland). The cell suspension was subjected to ultrasonication, followed by centrifugation to remove debris. The resulting supernatant was incubated overnight at 4°C with primary antibodies and protein G agarose beads (Roche, 11243233001, Switzerland).

For endogenous co‐IP, TBEV‐infected SH‐SY5Y cells (MOI = 2.0, 40 h) were exposed to 10 µm MG132 for 6 h, followed by harvesting and lysis in NP‐40 buffer (50 mm Tris‐HCl pH 7.5, 150 mm NaCl, 1% NP‐40, 5% glycerol) containing MG132 (10 µm) and protease inhibitors. Clarified lysates (2 mg of protein) obtained after centrifugation (13 000 × *g*, 15 min, 4°C) were incubated with 4 µg of the indicated primary antibodies or their corresponding isotype control IgGs (Cell Signaling Technology; #2729 for pAb, and #3900 for mAb) overnight at 4°C, followed by an additional 4 h incubation with Protein G agarose beads. Proteins were then eluted by boiling in 1× SDS loading buffer and analyzed by standard IB using the indicated antibodies. For both IP assays, the bead‐immune complexes were washed 6–8 times with washing buffer at 4°C. Proteins were eluted by adding 1× SDS‐loading buffer and boiling at 100°C for 10 min. Immunoprecipitated proteins and lysates were analyzed by IB.

The following primary antibodies were employed in this study: rabbit anti‐RNF138 polyclonal antibody (pAb) (ABclonal, A10304, China), mouse anti‐β‐Actin monoclonal antibody (mAb) (GenScript, A00702, China), mouse anti‐GAPDH mAb (Proteintech, 60004‐1‐lg, USA), rabbit anti‐TBEV NS1 mAb (Genetex, GTX642145, USA), rabbit anti‐TBEV NS5 mAb (Genetex, GTX642476, USA), rabbit anti‐ZIKV NS1 mAb (Genetex, GTX638945, USA), rabbit anti‐Myc pAb (Proteintech, 16286‐1‐AP, USA), mouse anti‐Myc mAb (Proteintech, 600003‐2‐Ig, USA), mouse anti‐HA mAb (Biolegend, 901514, USA), mouse anti‐Flag mAb (Sigma, F1804, USA), rabbit ubiquitin pAb (Santacruz, SC‐8017, USA), rabbit anti‐GST Tag pAb (BBI, D110271‐0100, China), and mouse anti‐6×His‐Tag mAb (BBI, D110002‐0100, China).

### 4.6 Proximity Labeling and Mass Spectrometry (MS)

To identify proteins interacting with NS5, we employed proximity‐dependent biotin labeling using the TurboID system [29]. HEK293T cells expressing Flag‐TurboID‐NS5 fusion protein were cultured for 18–24 h, treated with 10 µm MG132 or DMSO for 12 h, and then labeled with 500 µm biotin (Sigma, B4639, USA) for 10 min at 37°C. The labeling reaction was terminated by placing cells on ice, followed by five washes with ice‐cold PBS. Cells were gently detached using cold PBS and collected by centrifugation at 1500 × *g* for 3 min at 4°C. Cell pellets were lysed in RIPA buffer (50 mm Tris‐HCl pH 8.0, 150 mm NaCl, 0.1% SDS, 0.5% sodium deoxycholate, 1% Triton X‐100) supplemented with protease inhibitors and 1 mm PMSF. Lysates were clarified by centrifugation at 10 000 × *g* for 10 min at 4°C, repeated twice. For biotinylated proteins enrichment, clarified lysates were incubated with streptavidin magnetic beads (MedChemExpress, HY‐K0208, USA) overnight at 4°C with rotation. Beads were washed sequentially with: RIPA buffer (twice), 1 m KCl, 0.1 m Na_2_CO_3_, 2 m urea in 10 mm Tris‐HCl (pH 8.0), and finally RIPA buffer (twice). Bound proteins were eluted by boiling in Laemmli buffer containing 20 mm DTT and 2 mm biotin, separated by SDS‐PAGE, and subjected to in‐gel tryptic digestion. Resulting peptides were analyzed by MS.

### 4.7 Ubiquitin Remnant Profiling

For ubiquitin remnant profiling, HEK293T cells were co‐transfected with Flag‐tagged TBEV‐NS5 and 6×His‐ubiquitin. As described above, following MG132/DMSO treatment, Flag‐NS5 was immunoprecipitated using anti‐Flag M2 affinity gel (Sigma, A2220, USA) and competitively eluted using Flag peptide (MedChemExpress, HY‐P0319, USA). Ubiquitinated NS5 bands were visualized by Coomassie staining, excised from gels, and digested with trypsin. Ubiquitin‐modified peptides were enriched using K‐ε‐GG antibody, followed by MS analysis.

### 4.8 Stable Cell Lines

To establish stable RNF138‐silenced cell lines, the RNF138 gene was cloned into the pLKO.1‐puro vector (Addgene, 8453, USA). To generate RNF138‐overexpressing cells, the RNF138 gene was cloned into the pLVX‐IRES‐neo vector (BD Biosciences Clontech, 632164, USA). To produce lentiviral particles, HEK293T cells were transfected with Lipofectamine 3000 Reagent (Invitrogen, L3000‐008, USA) along with the appropriate plasmids, including RRE, REV, VSV‐G, and the control vector/shRNF138‐pLKO.1 (or pLVX‐RNF138). The lentiviral supernatant, collected 48 h post‐transfection, was used to infect target cells for 2 d. Following infection, stable cell lines were selected using 1 µg mL^−1^ puromycin (Selleckchem, S7417, USA), and the successful expression of RNF138 was confirmed by IB. The shRNA and cloning primer sequences were listed in Table S3.

### 4.9 Immunofluorescence (IF)

IF signals were detected using species‐matched Alexa Fluor‐conjugated secondary antibodies from Thermo Fisher Scientific where appropriate: goat anti‐mouse IgG/Alexa Fluor 488 (A‐11001), donkey anti‐rat IgG/Alexa Fluor 488 (A‐21208), and goat anti‐rabbit IgG/Alexa Fluor 568 (A‐11011).

For cell IF, HeLa cells grown on glass‐bottom dishes (NEST, 801001, China), were processed 48 h post‐transfection. Cells were first fixed with 4% paraformaldehyde (PFA) at 37°C for 15 min. After 3 washes with PBS, permeabilization was carried out using 0.2% Triton X‐100 at 37°C for 5 min, and were blocked by incubation with 10% FBS for 1 h at 24°C. Cells were then probed with appropriate primary antibodies overnight at 4°C. After washing, cells were exposed to specified secondary antibodies for 1 h at 24°C in darkness. Nuclear staining was achieved with DAPI (Sigma, D9542, USA). All images were captured using a FV3000 confocal microscope (Olympus, FV3000, Japan).

For tissue IF, paraffin‐embedded mouse brain sections (5 µm) were deparaffinized, rehydrated, and subjected to antigen retrieval by heating in citrate buffer (pH 6.0). After cooling and washing with PBS, sections were permeabilized with 0.2% Triton X‐100 for 10 min and blocked with 10% FBS for 1 h at 24°C. For co‐localization analyses, sections were incubated overnight at 4°C with rat anti‐Myc mAb (Abcam, ab206486, UK) together with either rabbit anti‐CD31 pAb (Proteintech, 28083‐1‐AP, USA) or rabbit anti‐NeuN pAb (Proteintech, 26975‐1‐AP, USA). After washing, sections were incubated with the appropriate secondary antibodies for 1 h at 24°C in the dark. Nuclei were counterstained with DAPI. Coverslips were mounted with anti‐fade mounting medium. Images were acquired using an Olympus VS200 slide scanning system.

### 4.10 FRET Analysis

HeLa cells were cultured in glass‐bottom dishes (NEST, 801001, China). The cells were co‐transfected with 1 µg of YFP‐NS5 and 1 µg of CFP‐RNF138. After 48 h of transfection, the cells were fixed with 4% PFA in PBS at 37°C for 15 min and washed three times with PBS.

Fluorescence imaging of the cells was performed using the FV 3000 confocal microscope system (Olympus, FV3000, Japan). FRET efficiency was quantified by measuring the fluorescence intensity of the donor CFP‐RNF138 (before and after bleaching) and the acceptor YFP‐NS5 channels within the regions of interest (ROIs). Image analysis was conducted using ImageJ software to quantify the fluorescence intensities, and FRET efficiency was obtained based on the donor fluorescence changes following acceptor photobleaching.

### 4.11 RNA Extraction and Real‐Time Quantitative PCR (qPCR)

The RNA was isolated from cultured cells and murine tissues using Trizol Reagent (Epizyme Biotech, YY101, China). The RNA reverse transcription was then performed using a cDNA Reverse Transcription kit (Applied Biosystems, 4368814, USA). Finally, qPCR was performed using UltraSYBR Mixture (CWBIO, CW3360M, China) and gene‐specific primers in a 20 µL reaction volume on an Mx3005P Real‐Time PCR System (Agilent Technologies, Mx3005P, USA). The amplification protocol consisted of an initial denaturation step at 95°C for 2 min, followed by 40 cycles of denaturation at 95°C for 30 s, primer annealing at 55°C for 30 s, and extension at 72°C for 30 s. Specific primers used are detailed in Table S4. The mRNA expression data were first normalized to the control group, and the relative fold changes were then derived using the 2^(−ΔΔCt)^ calculation.

### 4.12 Protein Purification and Pulldown Assays

*E. coli BL21(DE3)* (TransGen Biotech, CD601‐02, China) cells were transformed with plasmids encoding dual 6×His‐tagged RNF138 or encoding a C‐terminal GST tagged RdRp domain of NS5. Selection of transformants was carried out on LB agar containing ampicillin (50 µg mL^−1^). Single colonies were inoculated into LB medium and cultured at 37°C until the OD_600_ reached 0.6–0.8. For protein induction, cultures were supplemented with 0.5 mm IPTG and then incubated at 16°C for over 16 h (100 rpm shaking). Cells were harvested by centrifugation (4000 × *g*, 10 min, 4°C) and resuspended in Lysis Buffer A (20 mm Tris‐HCl pH 8.0, 1 mm protease inhibitor, 50 mm NaCl, 10 mm imidazole). Cell disruption was performed by ultrasonication on ice (20% amplitude, 3 s pulse on/3 s pulse off, total duration 10 min). Lysates were clarified by centrifugation (12 000 × *g*, 30 min, 4°C). For 6×His‐RNF138 purification, cleared lysates were incubated with Ni‐NTA agarose (Qiagen, 30210, Germany) pre‐equilibrated with Buffer A for 2 h at 4°C. Beads were washed with wash buffer (20 mm Tris‐HCl pH 8.0, 50 mm NaCl, 20 mm imidazole), and proteins were eluted with elution Buffer (20 mm Tris‐HCl pH 8.0, 250 mm imidazole, 50 mm NaCl). For NS5‐RdRp‐GST purification, cleared lysates were incubated with Glutathione Sepharose 4B (Cytiva, 17075601, USA) for 2 h at 4°C. Following extensive washing with PBS, bound proteins were then released by elution with a buffer containing 10 mm reduced glutathione in 50 mm Tris‐HCl, pH 8.0. Following elution, the purified TBEV‐RdRp‐GST and 6×His‐RNF138 proteins were concentrated and buffer‐exchanged into Buffer A using Amicon Ultra‐30 kDa (Merck Millipore, UFC903024, USA) and Amicon Ultra‐10 kDa (Merck Millipore, UFC901024, USA) centrifugal filter units, respectively.

For the GST pull‐down assay, purified 6×His‐RNF138 was incubated with either GST‐tagged NS5‐RdRp or GST alone (as a negative control) in binding buffer (20 mm Tris‐HCl, pH 7.5, 100 mm NaCl, 0.1% NP‐40, 1 mm DTT) for 2 h at 4°C with gentle rotation. GST complexes were then captured by incubation with Glutathione Sepharose beads for an additional 2 h at 4°C. The beads were collected and washed six times with 1 mm GSH buffer. Bound proteins were eluted by boiling the beads in 1× SDS loading buffer at 100°C for 10 min. Eluted proteins were detected by Coomassie blue staining and analyzed by IB.

### 4.13 Dual‐Luciferase (Luc) Reporter Assays

A549 cells were co‐transfected with the ISRE firefly Luc reporter, a Renilla Luc internal control plasmid (1 ng), and the indicated expression plasmids or empty vector. At 24 h post‐transfection, cells were stimulated with IFN‐β (1000 U mL^−1^) (Proteintech, HZ‐1298, USA) for an additional 24 h. Firefly and Renilla Luc activities were sequentially measured using the Dual‐Luc Reporter Assay System (Promega, E1910) on a GloMax 20/20 Luminometer (Promega), following the manufacturer's protocol.

### 4.14 In Vitro Ubiquitination Assay

Purified 6×His‐RNF138, its catalytic mutant 6×His‐RNF138‐C18A/C54A, and TBEV RdRp‐GST proteins were used for the in vitro ubiquitination assay. Recombinant E1 (UBA1), E2 (UBC5c), and K48‐linked ubiquitin (Ub‐K48) were prepared as described previously [16]. Ubiquitination reactions were carried out in a total volume of 50 µL containing 50 mm Tris‐HCl (pH 7.5), 1 mm DTT, 0.5 mm ATP, 5 mm MgCl_2_, 100 nm E1, 100 nm E2, 1 µm RNF138, 2.5 µm Ub‐K48, and 1 µm TBEV RdRp‐GST. After incubation at 37°C for 2 h, the reaction products were subjected to IB with an anti‐K48‐linkage‐specific pAb (ABclonal, A3606, China) to detect K48‐linked ubiquitination.

### 4.15 Mice Experiment

Specific pathogen‐free (SPF) female BALB/c mice, aged 6–8 weeks and weighing 18–22 g, were obtained from Vital River Laboratory Animal Technology (Beijing, China) and maintained under sterile SPF conditions. All animal procedures complied with the Animal Welfare and Ethics Committee of Changchun Institute of Biological Products Co.,Ltd (Approval No. CCIBP202510‐01). Mice were randomly divided into 4 groups (*n* = 8 per group): Group 1 (Vehicle+Vector): Hydrodynamic injection of 40 µg empty vector control (VR1012, 40 µg, in 500 µL saline); Group 2 (Vehicle+Myc‐RNF138): Myc‐RNF138 plasmid (40 µg, in 500 µL saline); Group 3 (TBEV+Vector): Vector control (VR1012) + TBEV challenge; Group 4 (TBEV+Myc‐RNF138): Myc‐RNF138 plasmid + TBEV challenge. For TBEV challenge, mice in Groups 3 and 4 were intraperitoneally inoculated with 10^3^ PFU TBEV (strain Senzhang) [32] 24 h post‐plasmid injection. Body weight and clinical signs were recorded daily. Mice exhibiting severe neurological symptoms, such as tremor, hind limb paralysis, or body stiffness, corresponding to a clinical score approaching 5 [57] were humanely euthanized and sampled for subsequent analysis. All remaining mice were euthanized at 8 days post‐infection (dpi). All TBEV experiments were performed in BSL‐3 containment (Changsheng Biotech, Changchun, China; ACDP Level 3 certified). All animal experiments were conducted in compliance with the ARRIVE guidelines.

### 4.16 H&E Staining

Brain tissues were fixed with 4% PFA and processed into 5 µm‐thick paraffin‐embedded sections. These sections were deparaffinized, rehydrated, and subjected to H&E staining using standard procedures to assess mice brain inflammation.

### 4.17 Statistical Analysis

Data are presented as mean ± standard deviation (SD). Data analysis was performed using GraphPad Prism software (version 8.0). Statistical significance between two groups was determined using the Student's t‐test, while comparisons across multiple groups were performed by one‐way ANOVA followed by Tukey's post‐hoc test. A *p*‐value of less than 0.05 was considered statistically significant. Significance levels are denoted as *p < 0.05, **p < 0.01, and ***p < 0.001; “ns” indicates no significance. Detailed statistical tests for each experiment are provided in the corresponding Figure legends.

## Author Contributions

W.Y.Z. and J.L.S. conceptualized and designed the study, analyzed the data, and wrote the manuscript. J.L.S., W.J.Y., and H.Z. performed the experiments. W.Y.Z. and X.J. provided funding and revised the manuscript. J.L.S., W.J.Y., H.Z., S.L., and J.Y.G performed the validation. J.L.S. prepared the visualizations. S.L. and J.Y.G. provided research resources and platforms. W.J.Y. and X.J. supervised the project. All authors have reviewed and approved the final manuscript.

## Funding

This work was supported by funding from Prevention and Control of Emerging and Major Infectious Diseases‐National Science and Technology Major Project (2025ZD01904302), the National Natural Science Foundation of China (82341072 and 82272316), the National Key R&D Program of China (2023YFC2306603), the Key Laboratory of Molecular Virology, Jilin Province (20102209), and Bethune Project of Jilin University (2023B03).

## Conflicts of Interest

The authors declare no conflicts of interest.

## Supporting information




**Supporting File**: advs75991‐sup‐0001‐SuppMat.docx.

## Data Availability

The mass spectrometry data were deposited in the iProx repository (http://www.iprox.cn) with the accession number IPX0014518001. The data that support the findings of this study are available upon reasonable request.
