# Supplementary material for: RNF138‐Mediated Ubiquitination and Degradation of NS5 Restricts Tick‐Borne Encephalitis Virus Infection
Source: Adv Sci (Weinh). 2026 Jun 10:e75991. Online ahead of print. doi: 10.1002/advs.75991 (PMC13336813; doi:10.1002/advs.75991)
Supplement: Supplementary file 1 — Supporting File: advs75991‐sup‐0001‐SuppMat.docx. [file ADVS-9999-e75991-s001.docx]

# Supporting Information

**RNF138-mediated ubiquitination and degradation of NS5 restricts tick-borne encephalitis virus infection**

*Jialiang Sun^1#^, Weijing Yang ^1#^, Hao Zhou^1^, Shuai Li^2^, Xin Jiang^3*^, Jianyang Gu^2*^, and Wenyan Zhang^1*^*

This Supplementary file includes:

Figures S1 to S6

Tables S1 to S4


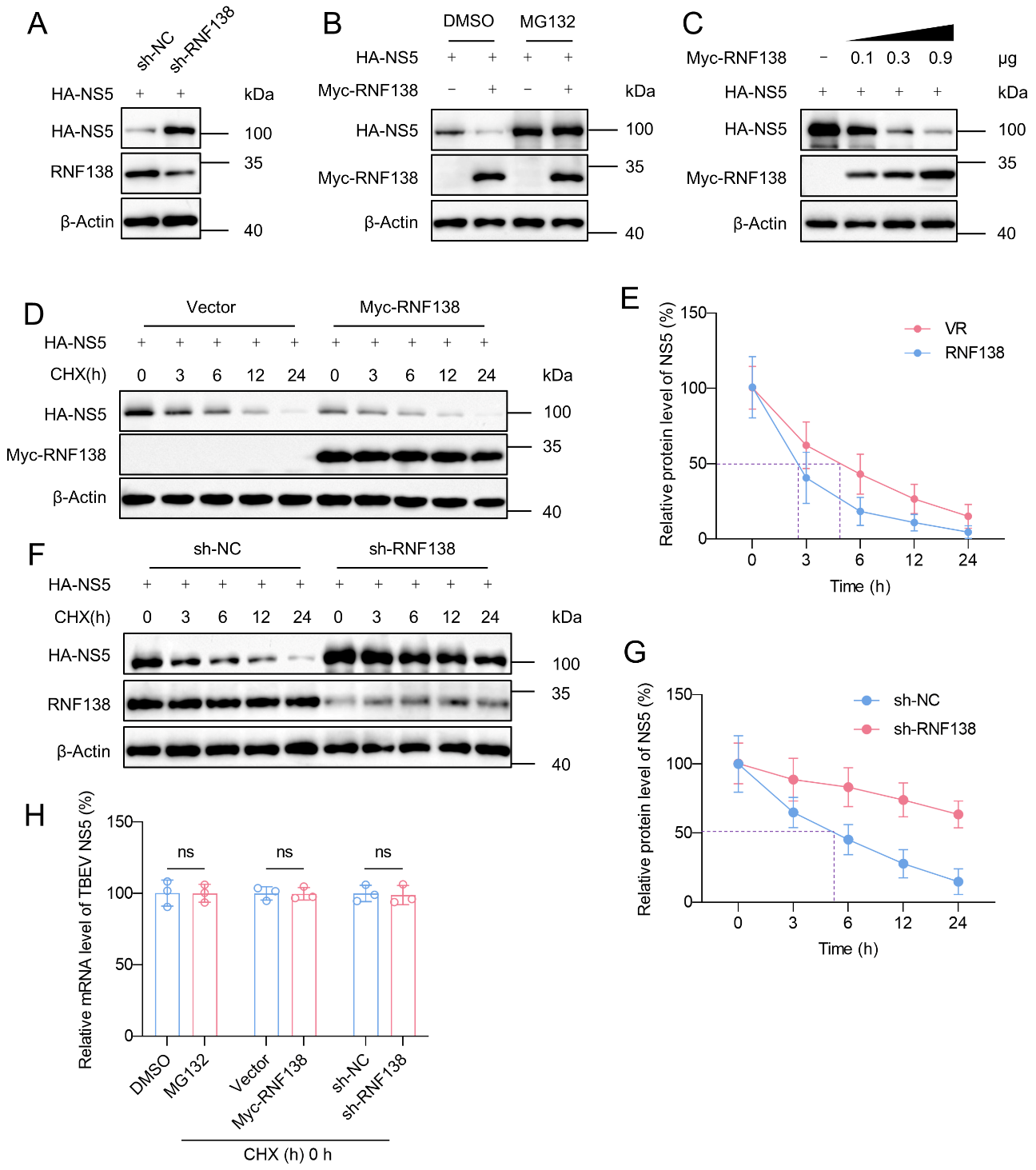


# Figure S1. RNF138 promotes TBEV NS5 degradation without affecting its mRNA level.

(A) MG132 stabilizes NS5 protein without altering its mRNA level. (B) Co-expression of RNF138 reduces NS5 protein abundance. HEK293T cells expressing HA-NS5 and Myc-RNF138 were treated with MG132 (10 μM, 12 h) before IB analysis. (C) RNF138 decreases NS5 levels in a dose-dependent manner. Cells were transfected with increasing amounts of Myc-RNF138 plasmid (0.1, 0.3, 0.9 μg) together with HA-NS5, followed by IB analysis. (D) RNF138 knockdown enhances NS5 stability. (E–F) RNF138 shortens NS5 half-life. (E) Cells expressing HA-NS5 and Myc-RNF138 were treated with CHX (50 μg/mL) and harvested at indicated time points for IB. (F) Quantification of NS5 levels normalized to β-Actin. (G–H) RNF138 knockdown prolongs NS5 protein half-life. (G) Control (shNC) or RNF138-knockdown cells expressing HA-NS5 were treated with CHX and collected at the indicated times. (H) Quantification of NS5 levels relative to β-Actin. (I) RNF138 does not affect NS5 mRNA expression. NS5 transcript levels were measured by RT-qPCR. Data are shown as mean ± SD from three independent experiments. ****P* < 0.001 by two-tailed t-test.


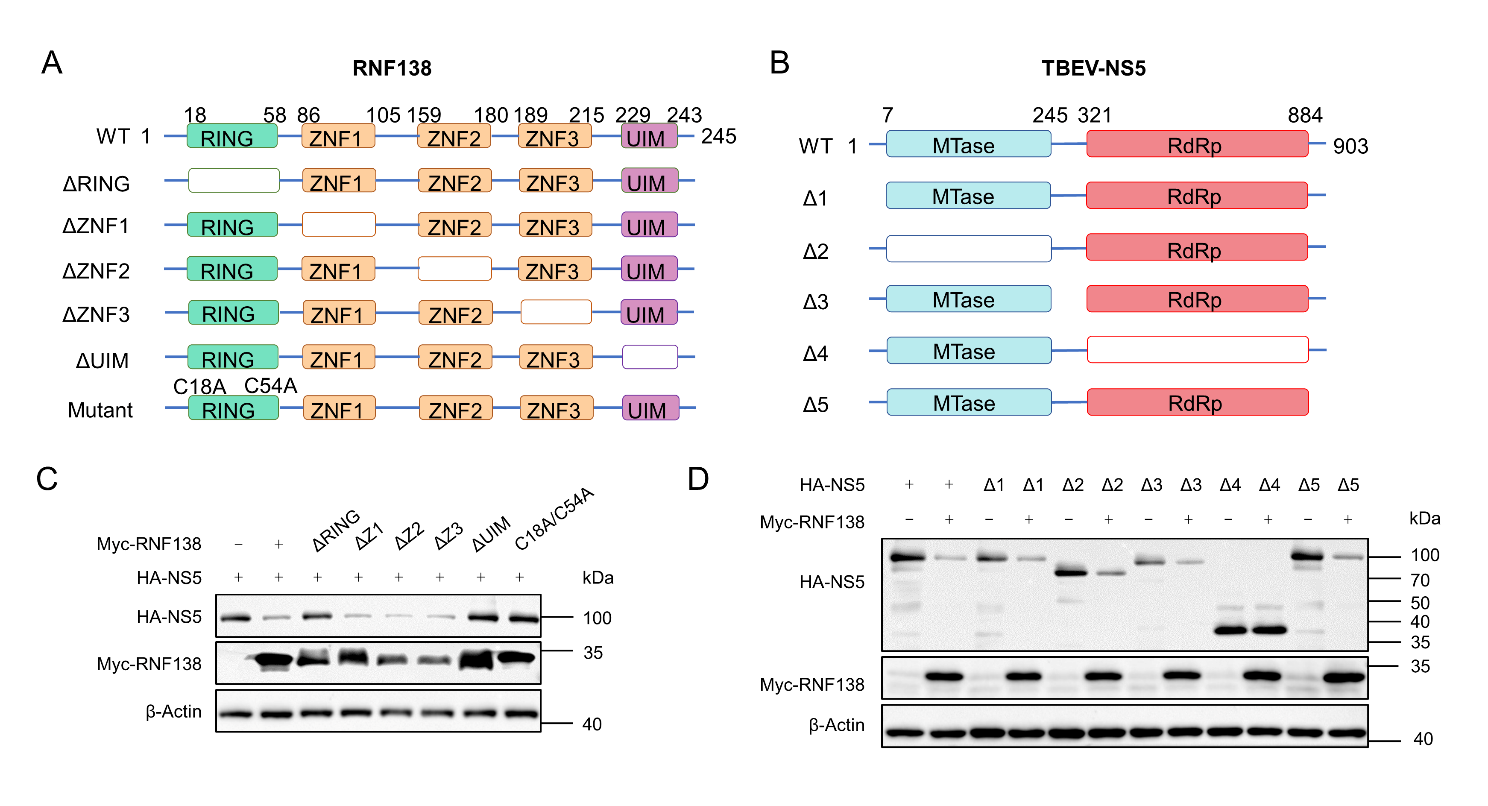


# Figure S2. RNF138 interacts with NS5.

(A) Domain architecture of human RNF138. RING: Really Interesting New Gene finger domain; ZNF1–3: zinc finger domains; UIM: ubiquitin-interacting motif. Numbers denote amino acid boundaries. (B) Schematic of NS5 deletion mutants used in this study. (C) The RING and UIM domains of RNF138 are essential for NS5 degradation. Cells expressing HA-NS5 with Myc-RNF138 truncation mutants were analyzed by IB at 48 h post-transfection. (D) The RdRp domain of NS5 is required for RNF138-mediated degradation. HEK293T cells expressing Myc-RNF138 and HA-NS5 truncation mutants were harvested at 48 h and analyzed by IB.


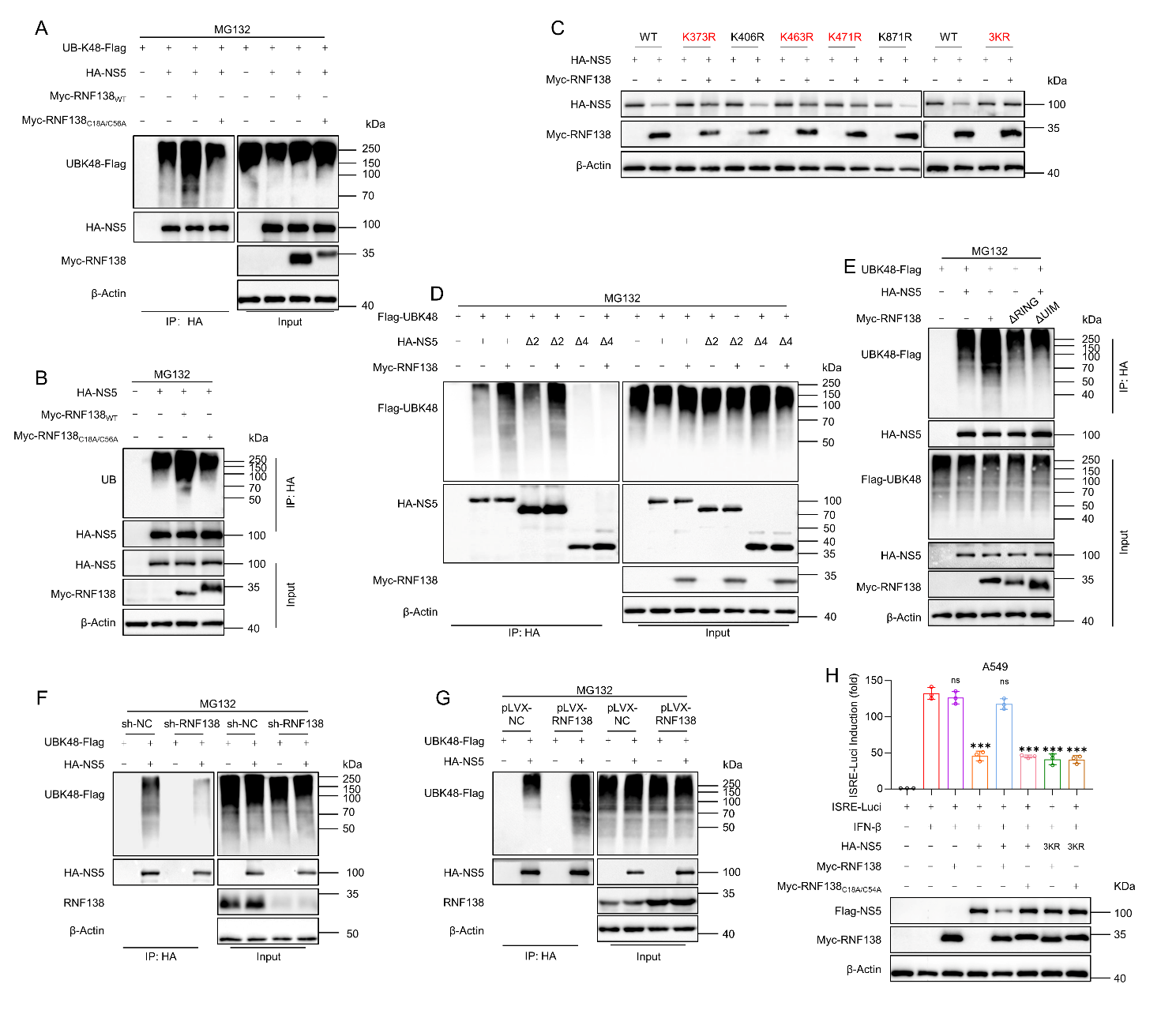


# Figure S3. RNF138 assembles K48-linked chains on NS5 and promotes its proteasomal degradation.

(A-B) RNF138 catalytic activity is required for K48-linked and total ubiquitination of NS5. (A) Cells co-expressing Ub-K48-Flag, HA-NS5, and Myc-RNF138 (WT or C18A/C54A) were analyzed by HA-IP and IB. (B) Cells co-expressing HA-NS5 and Myc-RNF138 (WT or catalytic mutant C18A/C54A) were analyzed by HA-IP and anti-ubiquitin IB. (C) Identified lysine residues are essential for RNF138-mediated NS5 degradation. Cells co-expressing Myc-RNF138 and HA-NS5 (WT or the indicated K to R mutants) were analyzed by IB for NS5 protein levels. (D) The RdRp domain of NS5 is necessary for RNF138-dependent ubiquitination. Cells co-expressing Myc-RNF138 and HA-NS5 (WT, Δ2, Δ4) were treated with MG132 and analyzed by HA-IP and IB. (E) The RING and UIM domains of RNF138 are essential for NS5 ubiquitination. Cells co-expressing HA-NS5 and Myc-RNF138 (WT, ΔRING, or ΔUIM) were treated with MG132 and analyzed by HA-IP and IB. (F) RNF138 knockdown reduces K48-linked ubiquitination of NS5. RNF138-silenced HEK293T cells expressing HA-NS5 and Ub-K48-Flag were subjected to HA-IP, followed by IB. (G) RNF138 overexpression enhances K48-linked ubiquitination of NS5. RNF138-overexpressing HEK293T cells expressing HA-NS5 and Ub-K48-Flag were analyzed by HA-IP and IB. (H) RNF138 attenuates NS5-mediated inhibition of IFN-β-induced ISRE promoter activation. A549 cells were transfected with ISRE-Luc reporter plasmid and Renilla luciferase reporter plasmid together with HA-NS5, HA-NS5-3KR, Myc-RNF138-WT, or the C18A/C54A mutant as indicated. At 24 h post-transfection, cells were stimulated with IFN-β (1000 U/mL) for another 24 h. ISRE-Luciferase reporter activity was measured and normalized to Renilla luciferase activity. Cell lysates were analyzed by IB. Data are presented as mean ± SD from three independent experiments. Statistical significance was determined by one-way ANOVA with Dunnett's post hoc test. ns, not significant; **P* < 0.05; ***P* < 0.01; ****P* < 0.001.


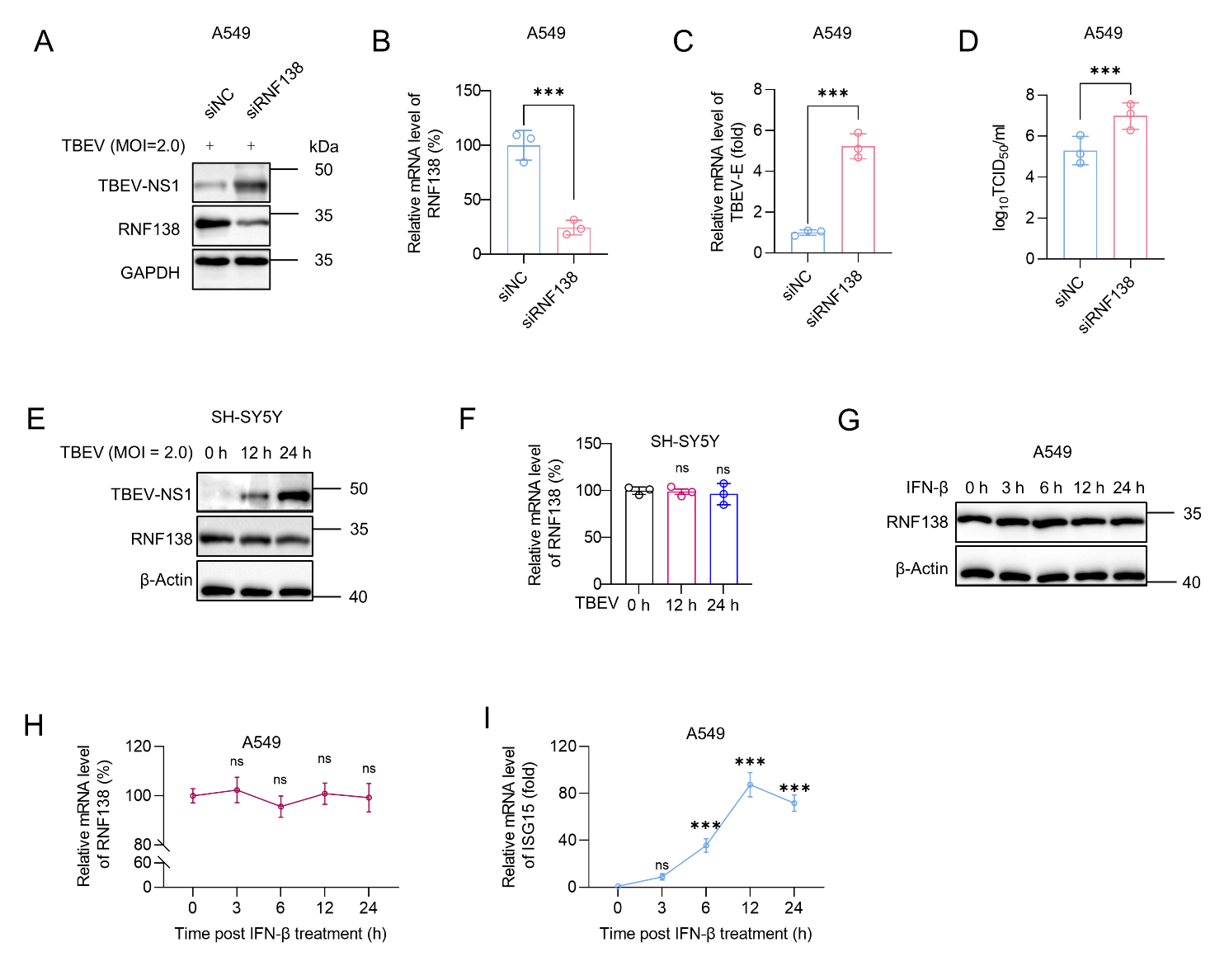


# Figure S4. RNF138 expression is not significantly altered by TBEV infection or IFN-β stimulation.

(A) A549 cells were transfected with RNF138-targeting siRNAs for 24 h, followed by TBEV infection (MOI = 2.0) for 48 h. TBEV NS1 protein was analyzed by IB. (B) Knockdown efficiency of RNF138 was confirmed by RT‑qPCR. (C) Viral RNA levels (TBEV-E gene) and (D) viral titers were assessed by RT‑qPCR and TCID_50_, respectively. (E–F) SH-SY5Y cells were infected with TBEV and harvested at 0, 12, and 24 h post-infection. (E) Endogenous RNF138 protein levels were analyzed by IB. (F) Intracellular RNF138 mRNA levels were measured by RT-qPCR. (G–I) A549 cells were treated with IFN-β (500 U/mL) for 0, 3, 6, 12, and 24 h. (G) Endogenous RNF138 protein levels were analyzed by IB. (H) RNF138 mRNA levels were measured by RT-qPCR. (I) ISG15 mRNA levels were measured by RT-qPCR as a positive control for IFN-β stimulation. Statistical significance was determined by two-tailed Student’s t-test (B-D), one-way ANOVA (F, I, J). Data are presented as mean ± SD; ns, not significant; **P* < 0.05; ***P* < 0.01; ****P* < 0.001. All data shown are representative of at least three independent experiments.


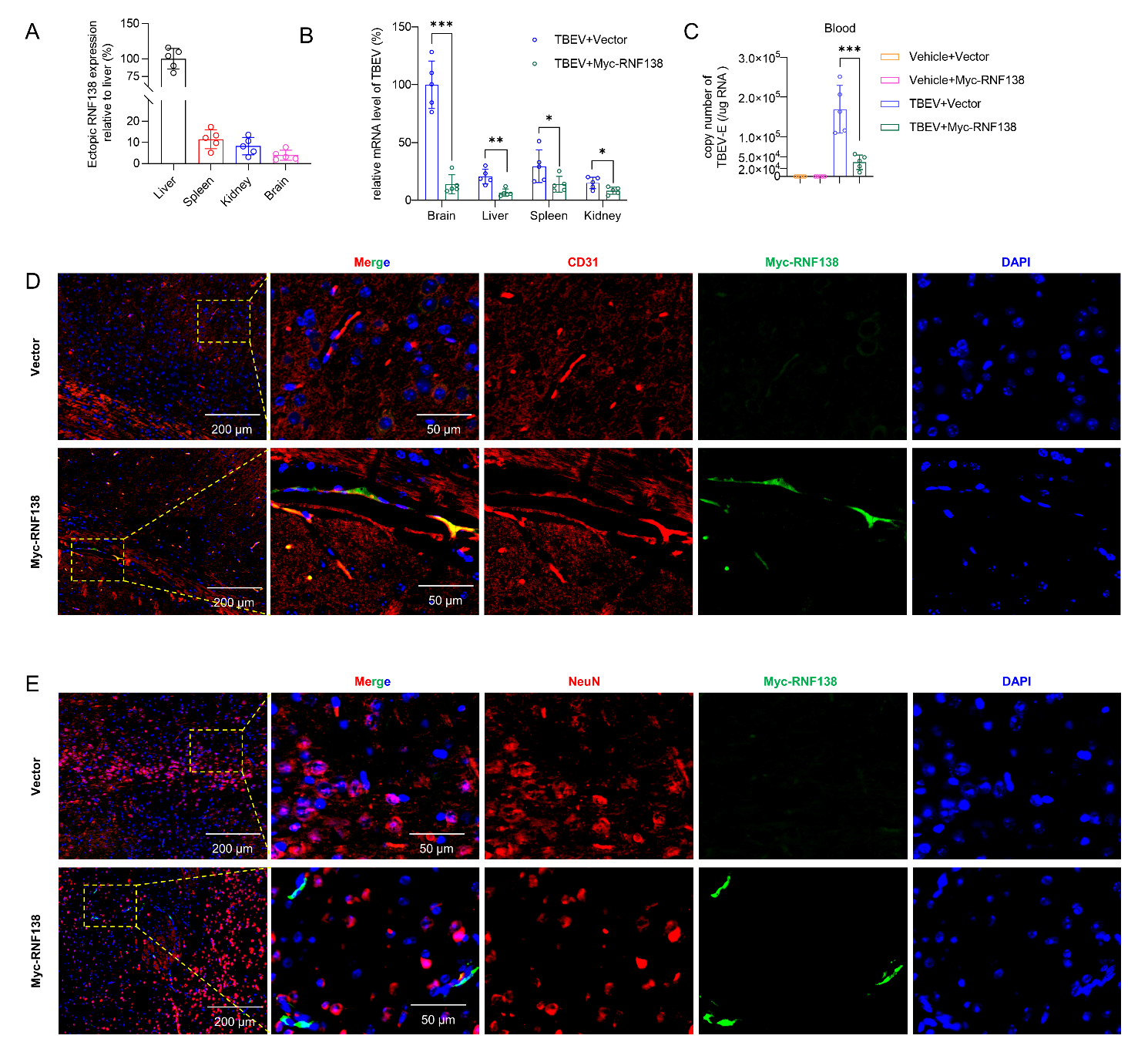


# Figure S5. Tissue distribution of ectopic RNF138 expression, TBEV burden, and brain localization of Myc-RNF138 in mice.

(A) Tissue distribution of ectopic Myc-RNF138 expression after hydrodynamic plasmid delivery. Vehicle + Vector and Vehicle + Myc-RNF138 mice were analyzed. Total RNA was extracted from the liver, spleen, kidney, and brain, and exogenous Myc-RNF138 transcript levels were determined by RT-qPCR using primers specific for the tagged construct. Expression levels were normalized to the internal control and are presented relative to the mean value of the liver sample in the Vehicle + Myc-RNF138 group, which was set to 100%. (B) Tissue distribution of TBEV burden in infected mice. TBEV + Vector and TBEV + Myc-RNF138 mice were analyzed. Total RNA was extracted from the brain, liver, spleen, and kidney, and Viral RNA levels (TBEV-E gene) levels were determined by RT-qPCR. Viral RNA levels were normalized to the internal control and are presented relative to the mean value of the brain sample in the TBEV + Vector group, which was set to 100%. (C) The viral RNA load (TBEV-E gene) in mice blood was determined by RT-qPCR. Data are presented as viral genome copies per microgram of total RNA (*n* = 5/group). (D–E) Cellular localization of ectopic Myc-RNF138 in mouse brain sections. Brain sections from Vehicle + Vector and Vehicle + Myc-RNF138 mice were subjected to double IF staining with anti-Myc together with (D) anti-CD31 or (E) anti-NeuN antibodies. Nuclei were counterstained with DAPI. Representative low-magnification images are shown with scale bars of 200 μm. Enlarged views are shown with scale bars of 50 μm, with merged images and individual Myc, CD31/NeuN, and DAPI channels displayed. Data in (A-C) are shown as mean ± SD. Each dot represents one mouse. Statistical significance was determined by unpaired two-tailed Student’s t-test (A, B), one-way ANOVA (C). ns, not significant; **P* < 0.05; ***P* < 0.01; ****P* < 0.001.


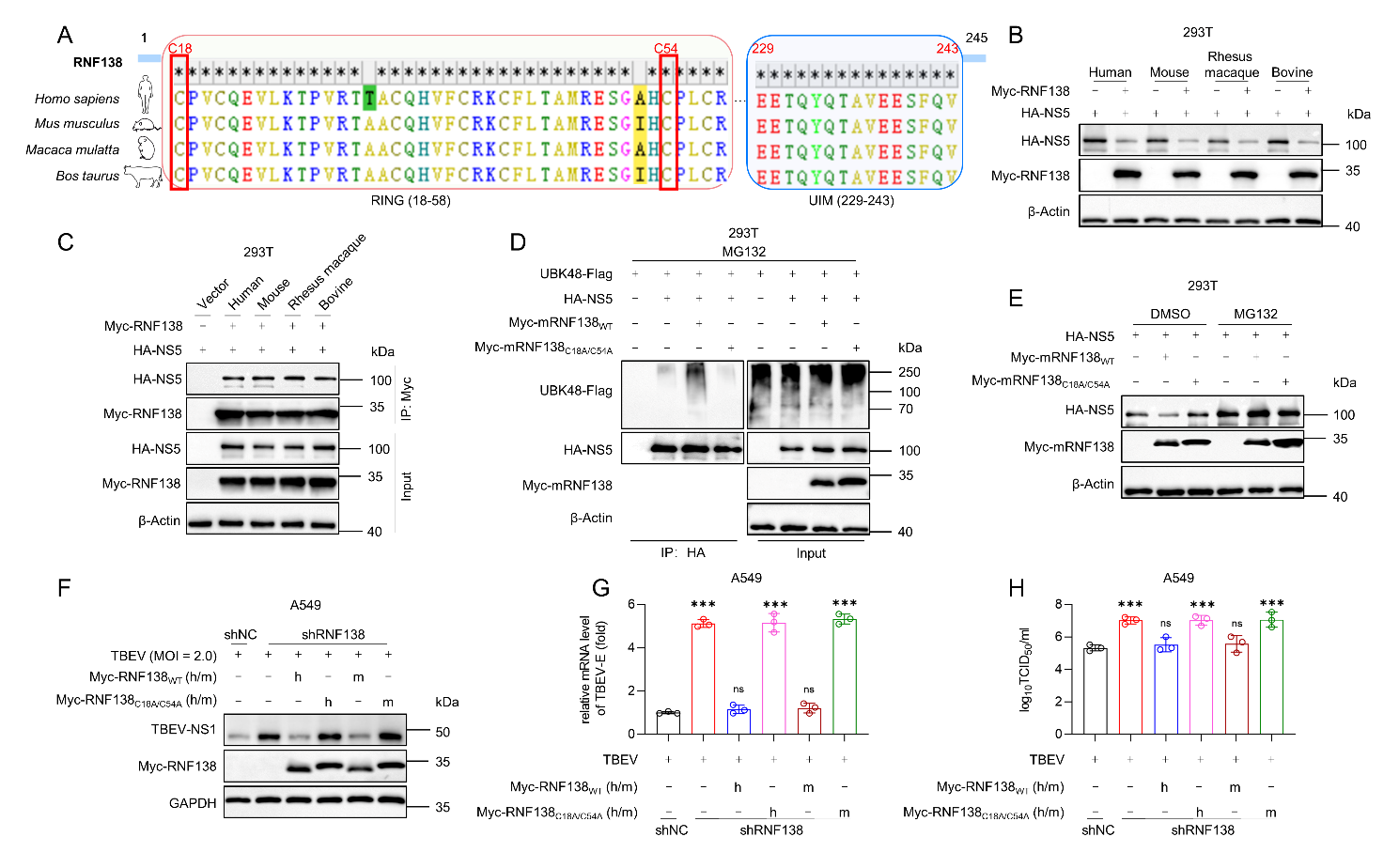


**Figure S6. RNF138-mediated recognition of TBEV NS5 and antiviral restriction are conserved across mammalian species.**

(A) Sequence alignment of RNF138 proteins from Homo sapiens, Mus musculus, Macaca mulatta, and Bos taurus. Conserved residues are shaded, and the catalytic cysteines in the RING domain (C18 and C54) are highlighted, additional details are provided in Table S2. (B) RNF138 proteins from several mammalian species reduce TBEV NS5 abundance. HEK293T cells were co-transfected with HA-NS5 together with Myc-RNF138 from the indicated species, and cell lysates were analyzed by IB. (C) RNF138 proteins from several different mammalian species interact with TBEV NS5. HEK293T cells were co-transfected with HA-TBEV NS5 and Myc-RNF138 from the indicated species. Cell lysates were subjected to Myc-IP, followed by IB. (D) Mouse RNF138 (mRNF138) promotes K48-linked ubiquitination of TBEV NS5 in a RING-dependent manner. HEK293T cells were co-transfected with HA-NS5, Ub-K48-Flag, and either Myc-mRNF138-WT or Myc-mRNF138-C18A/C54A. Cells were treated with MG132 before harvest, and lysates were subjected to HA-IP followed by IB. (E) The mRNF138-mediated degradation of TBEV NS5 depends on its catalytic activity and is reversed by proteasome inhibition. HEK293T cells were co-transfected with HA-NS5 together with Myc-mRNF138-WT or Myc-mRNF138-C18A/C54A, with or without MG132 treatment, and analyzed by IB. (F–H) Human and mouse RNF138 rescue antiviral activity in RNF138-deficient cells in a catalytic activity-dependent manner. sh-RNF138 A549 cells were reconstituted with shRNA-resistant human RNF138, mRNF138, or their corresponding catalytic mutants, followed by TBEV infection. (F) TBEV NS1 protein levels were analyzed by IB. (G) Viral RNA levels (TBEV-E gene) were measured by RT-qPCR. (H) Viral titers were determined by TCID_50_ assay. Data are shown as mean ± SD. Statistical significance was determined by two-tailed Student’s t-test. ns, not significant; **P* < 0.05; ***P* < 0.01; ****P* < 0.001. Unless stated otherwise, MG132 treatment = 10 µM, 12 h. All data shown are representative of at least three independent experiments.

# Table S1. The plasmids of flavivirus NS5 used in this study.

| Plasmid Name | Virus | Tag | Genebank ID |
| --- | --- | --- | --- |
| TBEV-Senzhang NS5-VR1012 | TBEV | HA | AY182009.1 |
| TBEV-Sib NS5-VR1012 | TBEV | HA | PV568692.1 |
| TBEV-Eur NS5-VR1012 | TBEV | HA | PV755118.1 |
| JEV NS5-VR1012 | JEV | HA | MH753128.1 |
| ZIKV NS5-VR1012 | ZIKV | HA | KU963796.1 |
| DENV NS5-VR1012 | DENV | HA | KY586735.1 |
| WNV NS5-VR1012 | WNV | HA | HM147822.1 |

# Table S2. RNF138 amino acid sequences used for vertebrate conservation analysis.

| Species | Common name | Protein name | UniProt accession number | Database |
| --- | --- | --- | --- | --- |
| *Homo sapiens* | Human | RNF138 | Q8WVD3 | UniProt |
| *Mus musculus* | Mouse | RNF138 | Q9CQE0 | UniProt |
| *Macaca mulatta* | Rhesus macaque | RNF138 | F6T5B4 | UniProt |
| *Bos taurus* | Bovine | RNF138 | Q32LN5 | UniProt |

# Table S3. The primers used for gene amplification, siRNA and shRNA.

| Primer Name | Sequence (5´ to 3´) | Purpose |
| --- | --- | --- |
| Flag-TurboID-EcoriⅠ-NS5-F | GCGAATTCGAGGCGGCAGCGAGGGC | Flag-TurboID-NS5 construction |
| XbaI-NS5-R | CGTCTAGAGATGATGCTGCTCTCCAG | Flag-TurboID-NS5 construction |
| NS5-Δ1-F | TTCCGGATTATGCAACCCTCGGCGAC | NS5-Δ1 truncation |
| NS5-Δ1-R | GTTGCATAATCCGGAACATCATACG | NS5-Δ1 truncation |
| NS5-Δ2-F | AGCGAGGGCGACTTCGGCGACCAAAG | NS5-Δ2 truncation |
| NS5-Δ2-R | AAGTCGCCCTCGCTGCCGCCTGC | NS5-Δ2 truncation |
| NS5-Δ3-F | GCTGGCCAGGTTCCTGATCAACGGCGT | NS5-Δ3 truncation |
| NS5-Δ3-R | AGGAACCTGGCCAGCAGCTTCCTG | NS5-Δ3 truncation |
| NS5-Δ4-F | AAGCGCTGCTAGCAGCTGCATGGACA | NS5-Δ4 truncation |
| NS5-Δ4-R | CTGCTAGCAGCGCTTCCTGTGGGA | NS5-Δ4 truncation |
| NS5-Δ5-F | GGTTCAAGGACTACTAAGGATCCAGATCT | NS5-Δ5 truncation |
| NS5-Δ5-R | TAGTAGTCCTTGAACCTCTCCTGGC | NS5-Δ5 truncation |
| NS5-K39R-F | ACCGAGAGGGACAGAGCTAGGGAGCTG | NS5-K39R mutant |
| NS5-K39R-R | CTGTCCCTCTCGGTCTCCAGGAT | NS5-K39R mutant |
| NS5-K62R-F | AGGGGCACAGCGAGGCTGGCGTGGCTTGAAGAGAG | NS5-K62R mutant |
| NS5-K62R-R | CTCGCTGTGCCCCTAGAAACCGCCAGGCCCATGT | NS5-K62R mutant |
| NS5-K74R-F | CTACGCCACACTTAGGGGCGAGGTGGT | NS5-K74R mutant |
| NS5-K74R-R | CTAAGTGTGGCGTAGCCCCTCTCT | NS5-K74R mutant |
| NS5-K108R-F | ACCATCGGAGGCAGGGGCCACGAGACA | NS5-K108R mutant |
| NS5-K108R-R | CTGCCTCCGATGGTGTAGGCCTT | NS5-K108R mutant |
| NS5-K160R-F | GTGGAAGGCGAGAGGACAAGGAAGGTGAT | NS5-K160R mutant |
| NS5-K160R-R | CTCTCGCCTTCCACCACAGCGTC | NS5-K160R mutant |
| NS5-K372R-F | GGAGCCCCAGCCAGGAACCAGGGTGATTATGAGGGC | NS5-K372R mutant |
| NS5-K372R-R | CTGGTTCCTGGCTGGGGCTCCTG | NS5-K372R mutant |
| NS5-K405R-F | GGAGTTCATCGCCAGGGTCAAGAGCAACGCCG | NS5-K405R mutant |
| NS5-K405R-R | CTGGCGATGAACTCCTCCCTGCTG | NS5-K405R mutant |
| NS5-K462R-F | CAAGAGGGAGAAGAGGCTGGGCGAGTTC | NS5-K462R mutant |
| NS5-K462R-R | CTCTTCTCCCTCTTGCCCATCATG | NS5-K462R mutant |
| NS5-K470R-F | TCGGCGTGGCCAGAGGAAGCAGGGCC | NS5-K470R mutant |
| NS5-K470R-R | CTGGCCACGCCGAACTCGCCCA | NS5-K470R mutant |
| NS5-K870R-F | GGAGCCGTGGAGAGGGTGAGGAAGATGA | NS5-K870R mutant |
| NS5-K870R-R | CTCTCCACGGCTCCCCAAATGTT | NS5-K870R mutant |
| XbaI-Flag- NS5-F | GCTCTAGAGCCACCATGGATTACAAGGACGACGATGACAAGGGCGGCAGCGAGGGC | Flag-NS5-VR1012 construction |
| BamHI-NS5-R | CGGGATCCTTAGATGATGCTGCTCTCC | Flag-NS5-VR1012 construction |
| XbaI-Myc-RNF138- F | GCTCTAGAATGGAACAGAAGCTTATCAGCGAGGAGGACCTTGCCGAGGACCTCTCTG | MYC-RNF138-VR1012 construction |
| BamHI-RNF138-R | CGGGATCCTCAGATGTTTACTTGAAAAGATTC | MYC-RNF138-VR1012 construction |
| RNF138-ΔRING-F | CGAAGATGATTTCTACCGTGGAAATGTGACT | RNF138-ΔRING truncation |
| RNF138-ΔRING-R | CGGTAGAAATCATCTTCGGTGTAGGAC | RNF138-ΔRING truncation |
| RNF138-ΔZ1-F | AAGTTTTCTGGTAGCAAGAAGTATCAGGATG | RNF138-ΔZ1 truncation |
| RNF138-ΔZ1-R | TTGCTACCAGAAAACTTCCTCATTAT | RNF138-ΔZ1 truncation |
| RNF138-ΔZ2-F | CATCCTACTTTTAAGTCTATTTCAGATAGTTC | RNF138-ΔZ2 truncation |
| RNF138-ΔZ2-R | AGACTTAAAAGTAGGATGACCAGAAGA | RNF138-ΔZ2 truncation |
| RNF138-ΔZ3-F | ATAGTTCCTGTGACACAATTTGATTATGGAG | RNF138-ΔZ3 truncation |
| RNF138-ΔZ3-R | TGTGTCACAGGAACTATCTGAAATAG | RNF138-ΔZ3 truncation |
| RNF138-ΔUIM-F | TGGAGAATTTGTGAATAACATCTGAGGATCC | RNF138-ΔUIM truncation |
| RNF138-ΔUIM-R | TTATTCACAAATTCTCCATAATCAAAT | RNF138-ΔUIM truncation |
| RNF138-C18A-F | GAAGATGATTTCTACGCCCCCGTCTGTCAG | RNF138-C18A mutant |
| RNF138-C18A-R | GTCCTACACCGAAGATGATTTCTACG | RNF138-C18A mutant |
| RNF138-C54A-F | AAAGCGGAGCACATGCTCCCCTATGTCGTG | RNF138-C54A mutant |
| RNF138-C54A-R | GCATGTGCTCCGCTTTCCCTCATTG | RNF138-C54A mutant |
| pGEX-TBEV-RdRp-F | GAGATATACACATGGGATCCCTGATCAACGGCGTGGTGAA | recombinant protein expression in *Escherichia coli* |
| pGEX-TBEV-RdRp-R | CCGTCGACGAGGTACCCAGGTAGTCCTTGAACCTCTC | recombinant protein expression in *Escherichia coli* |
| pET28-RNF138-F | ACCTGTACTTCCAGGGATCCGCCGAGGACCTCTCTGC | recombinant protein expression in *Escherichia coli* |
| pET28-RNF138-R | CCGTCGACGAGGTACGATGTTTACTTGAAAAGATTCTTCA | recombinant protein expression in *Escherichia coli* |
| RNF138-Ecori-pLVX-F | GAATTCATGGCCGAGGACCTCTCT | RNF138-overexpressing cell lines |
| RNF138-BamHⅠ-pLVX-R | GGATCCTCAGATGTTTACTTGAAAAGATTCT | RNF138-overexpressing cell lines |
| sh-RNF138-pLKO.1-F | CCGGCCCTGTGTCAAGAATCAAATTCTCGAGAATTTGATTCTTGACACAGGGTTTTTG | RNF138-silenced cell lines |
| sh-RNF138-pLKO.1-R | AATTCAAAAACCCTGTGTCAAGAATCAAATTCTCGAGAATTTGATTCTTGACACAGGG | RNF138-silenced cell lines |
| si-RNF138 | GCUAGAUGAAGAAACCCAAUA | Shutting down RNF138 |
| si-LTN1 | GCAUUUCAUCUGAUGAAGUAA | Shutting down LTN1 |
| si-RBBP6 | GCAAAGUCAUAGAAGUCGAAA | Shutting down RBBP6 |
| si-RNF185 | CCUCUUCUGUUGGCCGUGUUU | Shutting down RNF185 |
| si-MIB1 | UUGACAUUGGUAUUAUCCUUG | Shutting down MIB1 |
| si-UHRF1 | CGAGAUCUUUCCGGCAACA | Shutting down UHRF1 |
| si-TRIP12 | GGGCCAUGUUAGAAAUCCA | Shutting down TRIP12 |
| si-MID1 | UUUGCUUCAAUUUGUCAUAGC | Shutting down MID1 |

# Table S4. The primer sequences for RT-qPCR.

| Primer Name | Sequence (5´ to 3´) |
| --- | --- |
| GAPDH-homo-RT-F | CCCATCACCATCTTCCAGG |
| GAPDH- homo-RT-R | TTCTCCATGGTGGTGAAGAC |
| LTN1-homo-RT-F | GATGAGGCAGTCTCTTCCTATGC |
| LTN1-homo-RT-R | GCTGAAGTACACAAATGGCTGGG |
| RNF138-homo-RT-F | CAGACAGCGTTTACTGGATCAC |
| RNF138-homo-RT-R | TGGTAATCTGGCTAGGATCTCC |
| RBBP6-homo-RT-F | CTCCCCATACACTTCCTCTCC |
| RBBP6-homo-RT-R | TTCTTTTAGTCGTCGCTGCTC |
| RNF185-homo-RT-F | CTGTCACGCCTCTTCCTATTTGT |
| RNF185-homo-RT-R | GCCCAGCATTAGGCAATCAG |
| MIB1-homo-RT-F | ACTGGCAGTGGGAAGATCAA |
| MIB1-homo-RT-R | CATATGCTGCGCTATGTGGG |
| UHRF1-homo-RT-F | CCACACCGTGAACTCTCTGTC |
| UHRF1-homo-RT-R | AGGCGCACATCATAATCGAAG |
| TRIP12-homo-RT-F | GGATGCTGTGAGCAGAGAGA |
| TRIP12-homo-RT-R | CATCTGATCCATCGTCATCG |
| MID1-homo-RT-F | ATGGCCGAGTCCTGGCTACTGTT |
| MID1-homo-RT-R | AACTGGATCGACTATCGCAACTTC |
| TBEV-NS5-RT-F | GTACATGGAGGGCGAACACA |
| TBEV-NS5-RT-R | CAACCTAGGGTTGTGAGCGT |
| TBEV-E-RT-F | CTACCGCTTGGCAGGTGCACA |
| TBEV-E-RT-R | CAGCACGCCGGTCTGGTCGCCCA |
| ZIKV-E-RT-F | CGAGGACAGGCCTTGACTTT |
| ZIKV-E-RT-R | TTTTGGCATGTGCGTCCTTG |
| ISG15-homo-RT-F | CGCAGATCACCCAGAAGATCG |
| ISG15-homo-RT-R | TTCGTCGCATTTGTCCACCA |
| GAPDH-mus-RT-F | CAAGGCCGAGAATGGGAAG |
| GAPDH-mus-RT-R | TCCATGGTGGTGAAGACAC |
| RNF138-mus-RT-F | TTCTACTGCCCTGTCTGTCA |
| RNF138-mus-RT-R | CGTTCCGGACATGCTCTTTC |
| IL-1β-mus-RT-F | TGTGGAGAAGCTGTGGCAG |
| IL-1β-mus-RT-R | CAGCAGGTTATCATCATCATC |
| TNF-α-mus-RT-F | CTCAAAGACAACCAACTAGTG |
| TNF-α-mus-RT-R | TGGTATGAGATAGCAAATCGG |
| IL-6-mus-RT-F | CACTTCACAAGTCGGAGGC |
| IL-6-mus-RT-R | TTTGTATCTCTGGAAGTTTCAG |
| Myc-hRNF138-RT-F | AGCGAGGAGGACCTTATGGC |
| Myc-hRNF138-RT-R | CAAGGTCTAAGGCCCGTTCA |
